# Psychotherapy for comorbid depression and somatic disorders: a systematic review and meta-analysis

**DOI:** 10.1017/S0033291721004414

**Published:** 2023-04

**Authors:** Clara Miguel, Eirini Karyotaki, Marketa Ciharova, Ioana A. Cristea, Brenda W.J.H. Penninx, Pim Cuijpers

**Affiliations:** 1 Department of Clinical, Neuro and Developmental Psychology, Amsterdam Public Health Research Institute, Vrije Universiteit Amsterdam, The Netherlands; 2 WHO Collaborating Centre for Research and Dissemination of Psychological Interventions, Vrije Universiteit Amsterdam, The Netherlands; 3 Department of Brain and Behavioral Sciences, University of Pavia, Italy; 4 IRCCS Mondino Foundation, Pavia, Italy; 5 Department of Psychiatry, Amsterdam UMC, Amsterdam Public Health Research Institute, Vrije Universiteit Amsterdam, The Netherlands

**Keywords:** Depression, somatic disorders, psychotherapy, somatic health, quality of life, psychological interventions, meta-analysis

## Abstract

**Background:**

The treatment of depression in patients with somatic disorders is crucial, given its negative impact on quality of life (QoL), functioning, and even on the somatic disease prognosis. We aimed to examine the most updated evidence on the effects of psychotherapy in patients with depression and somatic disorders, including HIV, oncological, cardiometabolic, and neurological disorders.

**Methods:**

We conducted a meta-analysis of 75 randomized trials (8209 participants) of psychotherapy for adults with somatic disorders and a diagnosis or elevated symptoms of depression. Outcomes included depression, QoL, somatic health-related outcomes, and mortality.

**Results:**

Psychotherapy significantly reduced the severity of depression at post-treatment across all categories of somatic disorders (Hedges'*g* = 0.65; 95% CI 0.52–0.79), with sustained effects at 6–11 months (*g* = 0.38; 95% CI 0.22–0.53) and at 12 months follow-up or longer (*g* = 0.13; 95% CI 0.04–0.21). Psychotherapy also showed significant effects on QoL (*g* = 0.26; 95% CI 0.17–0.35), maintained up to 11 months follow-up (*g* = 0.25; 95% CI 0.16–0.34). No significant effects were observed on the most frequently reported somatic health-related outcomes (glycemic control, pain), and neither on mortality. Heterogeneity in most analyses was very high, and only 29 (38%) trials were rated at low risk of bias (RoB).

**Conclusions:**

Psychotherapy may be an effective treatment option for patients with depression and somatic disorders, with long-term effects on depression severity and QoL. However, these results should be interpreted with caution due to heterogeneity and RoB.

## Introduction

Individuals with somatic disorders have an increased risk of experiencing depression (Egede, 2007; Moussavi et al., 2007). In this population, depression has been associated with significant decrements in quality of life (QoL) (Moussavi et al., 2007), higher healthcare utilization and costs (Egede, 2007), lower adherence to medical treatments (DiMatteo, Lepper, & Croghan, 2000), and an increased risk of medical complications and mortality (Lichtman et al., 2014; Pederson, Warkentin, Majumdar, & McAlister, 2016; Pinquart & Duberstein, 2010). Thus, successfully treating depression could have a significant impact on the QoL and somatic disease progression of these patients.

Psychological interventions have proven to decrease the symptoms of depression and increase QoL in general adults with depression, with sustained effects over the long-term (Cuijpers, Karyotaki, de Wit, & Ebert, 2020; Karyotaki et al., 2016; Kolovos, Kleiboer, & Cuijpers, 2016). Furthermore, recent evidence suggests that these interventions may have an effect on biological parameters, such as inflammatory biomarkers (O'Toole et al., 2018) and immune system function (Shields, Spahr, & Slavich, 2020).

In patients with comorbid depression and somatic disorders, previous meta-analytical studies have suggested that psychotherapy is an effective treatment option (Rizzo, Creed, Goldberg, Meader, & Pilling, 2011; van Straten, Geraedts, Verdonck-de Leeuw, Andersson, & Cuijpers, 2010). However, many more trials have been published over the last years on a range of somatic disorders, which could change previous conclusions. Moreover, the extent of psychotherapy effects on QoL, somatic health-related outcomes (e.g. cardiac events), or mortality is still uncertain.

We conducted a systematic review and meta-analysis to estimate the effectiveness of psychotherapy for patients with depression and somatic disorders. We examined outcomes covering depression, QoL, somatic health-related outcomes, and mortality, in patients with a wide range of somatic illnesses, including HIV/AIDS, oncological, cardiometabolic, and neurological disorders.

## Methods

### Identification and selection of studies

The protocol of this study was prospectively registered in Open Science Framework (https://osf.io/q6z3p). We used the most recent version of an existing database of randomized trials on psychotherapies for depression (https://osf.io/825c6). This database was developed through systematic searches in PubMed, PsycINFO, Embase, and Cochrane (from database inception to 1 January 2020), by combining index and free terms indicative of depression and psychotherapies (search string is provided in eMethods in the online Supplementary material). Two researchers screened and selected all records, solving disagreements through discussion.

For this systematic review and meta-analysis, we included: (1) randomized controlled trials (RCTs) (2) comparing psychological interventions (3) against control conditions (waiting-list, care-as-usual, other inactive treatment) (4) in adults with depression (5) and a comorbid somatic illness (e.g. diabetes, HIV, etc.). Depression could be established through a diagnostic interview or a cut-off on a validated self-report questionnaire. The somatic disorder could be acute or chronic. Any type of psychotherapy (cognitive-behavior therapy, ‘third wave’ therapies, supportive therapy, etc.) and different delivery formats (individual, group, and guided self-help) were included. We excluded studies on self-guided interventions without any professional support. Studies on inpatients or on bipolar and psychotic depression were excluded, as well as maintenance trials.

### Data extraction and risk of bias

We extracted data involving (1) characteristics of the studies (e.g. type of control, recruitment, type of psychotherapy, use of booster sessions after the treatment), (2) characteristics of the participants (e.g. diagnosis/symptoms of depression, type of somatic disorder, mean age), (3) study drop-out (due to any reason during the acute phase treatment), and (4) post-intervention and follow-up outcome data on depression, QoL, somatic health-related outcomes, and mortality.

In line with previous meta-analyses using our database of randomized trials, risk of bias (RoB) was assessed with five criteria of Cochrane's RoB tool (Higgins et al., 2011): (1) adequate generation of randomization sequence, (2) allocation concealment, (3) blinding of assessors, (4) appropriate methods for handling missing data (rated as positive for intention-to-treat analyses), and (5) selective outcome reporting (rated as positive when prospectively registered primary outcomes were consistently reported in the article). Items with lack of information were classified as high risk.

Two researchers performed data extraction and RoB assessment, solving disagreements by consensus or through discussion with a third researcher.

### Outcome measures

Effects were estimated for depression, QoL, somatic health-related outcomes, and mortality. Outcomes were extracted from primary and secondary publications of the same trial and comprised validated self-reports, interviews, or biological tests. An overview of all the extracted outcomes is provided in the online Supplementary eTable1.

*Depression* outcomes included any measure evaluating the severity of symptoms. When a study reported multiple instruments for measuring depression, we selected one based on a predefined algorithm (eMethods in the online Supplementary material).

*QoL* was defined as perceived physical and mental health status, well-being, and performance in daily life (Kolovos et al., 2016). Measures typically provided a total QoL score and/or separate scores for specific subcomponents, usually divided in mental and physical health-related QoL (e.g. SF-36). Following procedures from comparable research (Kolovos et al., 2016), we estimated the effects for *Overall QoL*, and also separately for *Physical* and *Mental QoL* subcomponents. When a study reported multiple measures, we selected the most frequently reported across studies. An overview of the QoL instruments used is presented in the online Supplementary eTable2.

*Somatic health-related outcomes* included measures assessing general somatic health status, common across disorders (e.g. inflammation biomarkers, pain, etc.) or specific to a category of disorder (e.g. cardiac events, HIV viral load, glycemic control, etc.). We examined those that were present in a minimum of five studies.

*Mortality* data comprised the number of participants that died —due to any cause— during the trial, from randomization until the last follow-up.

### Meta-analyses

We conducted separate meta-analyses for depression, QoL, each specific somatic health-related outcome, and mortality. Effects were estimated at post-treatment, and when available, at long-term follow-ups (from 6 months post-randomization).

For meta-analyses based on continuous outcomes, we calculated effect sizes (Hedges' *g*) for each comparison between a psychotherapy and control condition. We used means and standard deviations, and when these were not reported, dichotomous outcomes or other statistics (e.g. *p* value, *t* value). Effect sizes were pooled with a random-effects model, with a restricted maximum-likelihood estimator (Viechtbauer, 2005), and using the Hartung-Knapp-Sidik-Jonkman method (IntHout, Ioannidis, & Borm, 2014). We examined potential effect modifiers (e.g. study characteristics) in meta-regression and subgroup analyses using a mixed-effects model.

For meta-analyses based on dichotomous outcomes, we calculated odds ratios (OR). For mortality, OR were pooled using Peto's method (Yusuf, Peto, Lewis, Collins, & Sleight, 1985), indicated for outcomes with infrequent events and with similar numbers between arms. Additionally, we calculated OR for study drop-out and pooled with the Mantel-Haenszel method (Robins, Greenland, & Breslow, 1986), using a treatment arm continuity correction.

Heterogeneity was estimated with the *I*^2^ statistic and its 95% confidence interval (CI). We included prediction intervals (PI), which represent 95% CI of the predictive distribution of effects in future comparable trials. Publication bias was explored using Egger's test (Egger, Smith, Schneider, & Minder, 1997).

Sensitivity analyses were conducted by (1) excluding outliers (studies whose 95% CI effect size did not overlap with the 95% CI of the pooled effect), (2) limiting analyses to studies at low RoB (⩾4 items rated as low risk), and (3) adjusting for publication bias with Duval and Tweedie trim-and-fill procedure (Duval & Tweedie, 2000).

We used the Comprehensive Meta-analysis (CMA) (version 3.3070) to calculate the individual effect sizes, and R (version 3.6.2) to perform all the meta-analyses, using the packages *meta* (Balduzzi, Rücker, & Schwarzer, 2019) and *dmetar* (Harrer, Cuijpers, Furukawa, & Ebert, 2019).

## Results

### Selection and inclusion of studies

**Fig. 1.**
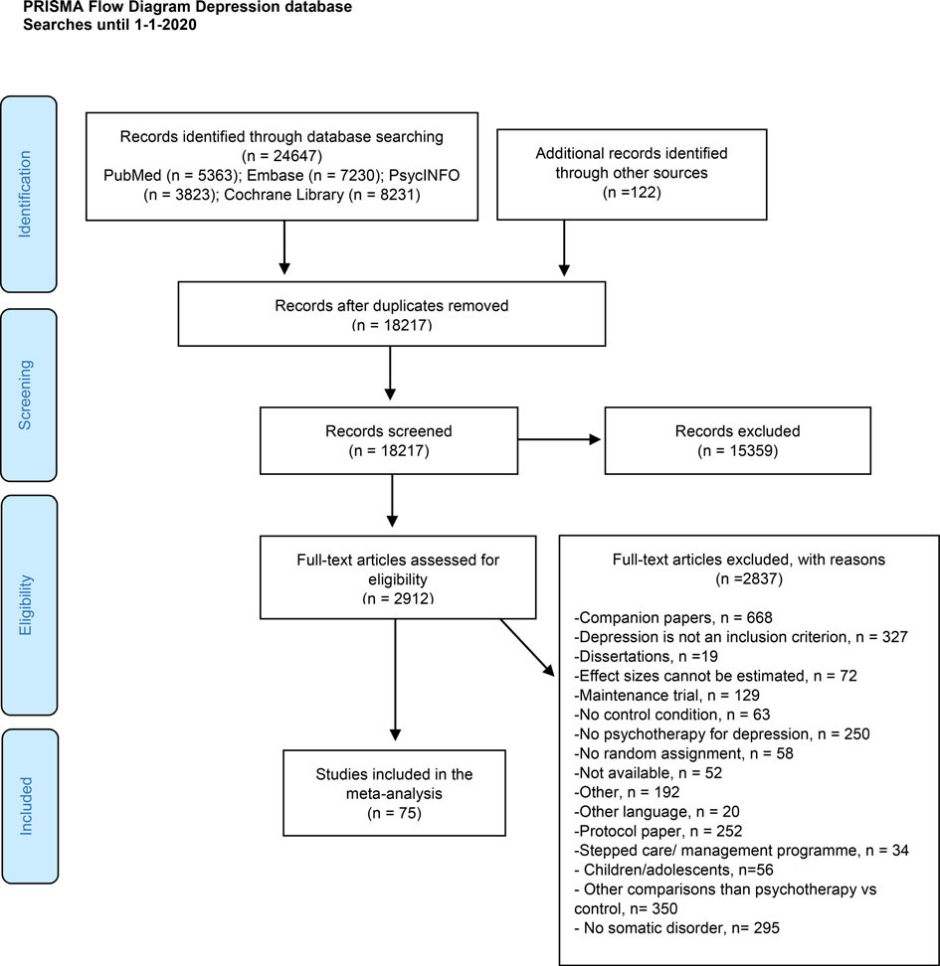
PRISMA flow diagram describing the selection and inclusion process.

The PRISMA flowchart describing the selection and inclusion process is presented in Fig. 1. We screened 24 769 abstracts (18 217 after removing duplicates) and examined 2912 full texts. A total of 75 RCTs met the criteria for inclusion. The reference list of included studies is provided in the online Supplementary material (eResults).

### Characteristics of included studies

Characteristics of the 75 included studies are presented in eTable3 (online Supplementary material). These trials included a total of 8209 participants, with 4437 in psychotherapy and 3772 in control conditions. Participants were mostly adults (*n* = 39 studies) or older adults (*n* = 35 studies) with elevated depression symptoms (*n* = 47 studies), recruited from medical settings (*n* = 45 studies) in Western countries (*n* = 61 studies). The most frequent type of psychotherapy was cognitive-behavioral therapy (*n* = 39 studies), delivered individually (*n* = 38 studies) or in a group (*n* = 27 studies), compared to care-as-usual (*n* = 48 studies).

The somatic disorders comprised a wide range of illnesses, which were classified based on type of disorder: (1) cardiometabolic disorders (*n* = 26 studies), including diabetes (*n* = 12 studies) and different types of cardiovascular disease (*n* = 14 studies); (2) HIV/AIDS (*n* = 13 studies); (3) oncological disorders (*n* = 11 studies), with many studies focusing on breast cancer (*n* = 5 studies); (4) neurological disorders (*N* = 10 studies), including, e.g. migraine or multiple sclerosis; and (5) other somatic disorders, which included those studied in a limited number of trials (*n* = 15 studies) (e.g. chronic pain, visual disorders, or heterogeneous samples of patients).

### Risk of bias

RoB was variable (online Supplementary eTable3). An adequate sequence generation was reported in 54 studies (72%), and 40 studies reported concealment of allocation (53%). Most of the trials used self-reports (*n* = 45 studies; 60%) or blind assessors (*n* = 25 studies; 33%), and applied intention-to-treat analyses (*n* = 51 studies; 68%). The vast majority of trials (*n* = 63 studies; 84%) were at risk of selective reporting, being most of them not registered (*n* = 28 studies) or retrospectively registered (*n* = 27 studies). In total, 29 (38%) trials were rated at overall low RoB.

### Study drop-out

Study drop-out was available for 79 comparisons between psychotherapy and control conditions: 848 (20%) drop-outs in the psychotherapy and 571 (15%) in the control groups. Pooling 72 trials with at least one drop-out for one of the conditions, we observed a significantly higher probability of drop-out for participants in the intervention groups (OR = 1.46, 95% CI 1.29–1.65; *I*^2^ = 0%; 95% CI 0–21). Drop-out ratios were further examined in a series of exploratory subgroup analyses of the most relevant study-level predictors (i.e. somatic disorders, control conditions, psychotherapies, and delivery formats). These analyses suggested significant differences for types of delivery formats (*p* = 0.03), with guided self-help interventions showing larger drop-out rates (OR = 1.94) than individual or group face-to-face treatments (OR = 1.32). None of the other predictors was significantly associated to drop-out.

### Effects of psychotherapy on depression severity

**Fig. 2.**
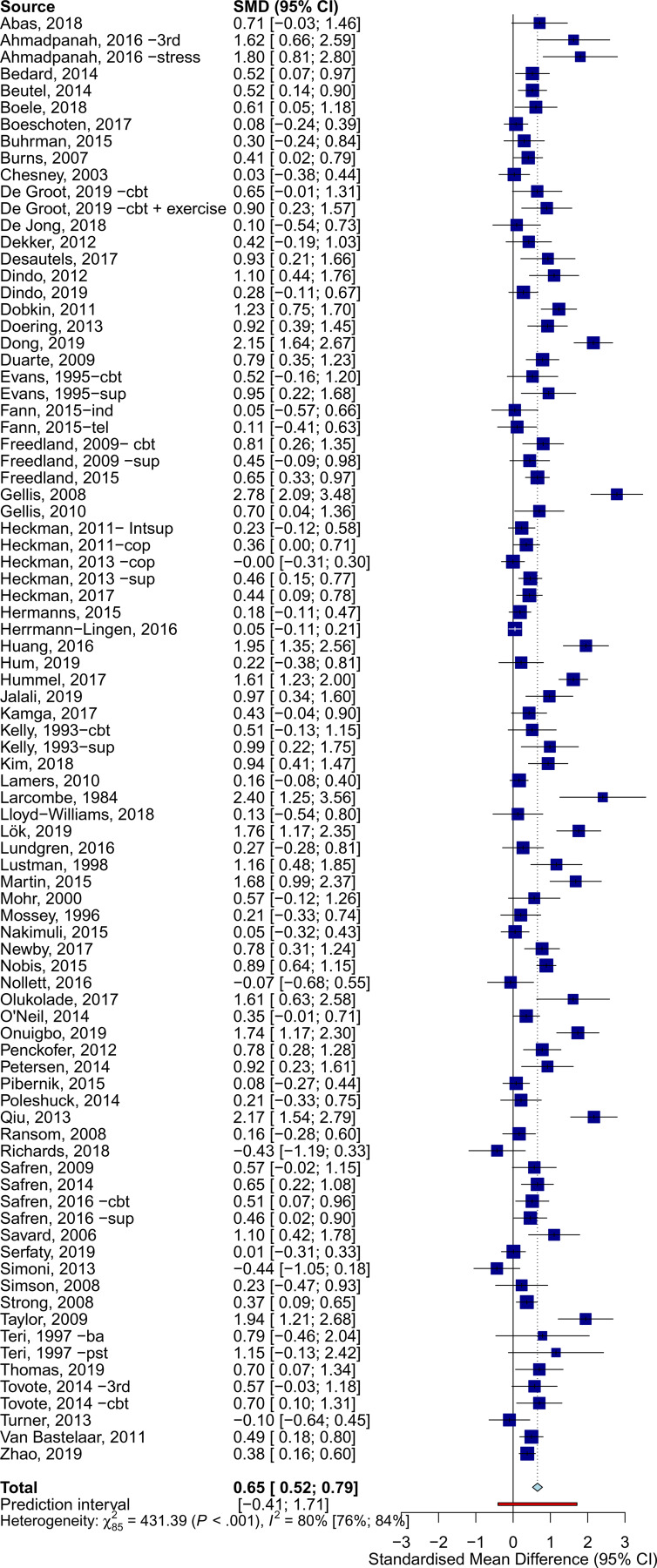
Effects of psychotherapy for depression across all types of somatic disorders.

The overall effects across all somatic disorders at post-treatment was *g* = 0.65 (95% CI 0.52–0.79) (Table 1, Fig. 2). Heterogeneity was very high (*I*^2^ = 80%, 95% CI 76–84), and PI included negative effects (−0.41 to 1.71). Egger's test suggested the presence of publication bias (*t* = 4.920, *p* < 0.001).

**Table 1.** Effects of psychotherapy on depression severity

	*N*_comp_	*g*	95% CI	*I*^2^	95% CI	*p* value^a^
All comparisons	86	0.65	0.52–0.79	80	76–84	NA
Cardiometabolic	30	0.75	0.51–0.99	83	76–88	*0.004*^b^
Cardiovascular	16	0.82	0.37–1.27	87	81–91	
Diabetes	14	0.69	0.42–0.95	72	52–84	
Oncological	12	0.85	0.46–1.25	87	78–92	
Breast	5	1.11	0.34–1.88	80	52–92	
Other	7	0.67	−0.01 to 1.34	88	79–94	
HIV/AIDS	17	0.34	0.19–0.50	45	3–69	
Neurological	11	0.96	0.53–1.38	83	70–90	
Other	16	0.48	0.23–0.74	67	67–87	
Subgroup analyses						
Age group						
Adults	45	0.64	0.48–0.81	72	62–79	0.893
Older adults	40	0.66	0.45–0.87	86	81–89	
Recruitment						
Community	34	0.55	0.38–0.72	69	56–78	0.201
Medical settings	51	0.72	0.53–0.90	84	80–88	
Diagnosis of depression						
Confirmed diagnosis	33	0.63	0.45–0.81	71	59–80	0.792
Elevated symptoms	53	0.66	0.48–0.84	84	79–87	
Type of psychotherapy						
CBT	42	0.75	0.56–0.93	81	74–85	0.122
PST	8	0.75	0.14–1.37	87	77–93	
SUP	8	0.40	0.23–0.58	16	0–59	
3rd wave	7	0.53	0.19–0.87	50	0–79	
Other	21	0.55	0.28–0.82	84	77–89	
Format						
Individual	38	0.64	0.45–0.84	74	65–81	0.187
Group	27	0.80	0.53–1.07	85	80–89	
Guided self-help	8	0.49	0.29–0.70	62	18–82	
Type of control						
Usual care	58	0.58	0.43–0.72	76	69–81	0.144
Waiting list	21	0.90	0.61–1.2	82	74–88	
Other	7	0.54	−0.03 to 1.1	90	82–94	
Country						
Western	71	0.52	0.40–0.64	74	67–79	*<0.001*
Non-Western	15	1.27	0.92–1.61	88	82–92	
Long-term outcomes						
6–11 months	46	0.38	0.22–0.53	75	67–81	NA
⩾12 months	13	0.13	0.04–0.21	0	0–57	NA
Sensitivity analyses						
Outliers excluded	63	0.55	0.47–0.62	28	1–48	NA
Studies at low RoB	31	0.54	0.33–0.75	83	77–88	NA
Adj. for publication bias	111	0.35	0.19–0.51	87	85–89	NA

*N*_comp_, number of comparisons; *g*, Hedges' *g*; PI, prediction intervals; CBT, cognitive-behavioral therapy; PST, problem-solving therapy; SUP, supportive therapy; 3rd wave, third wave therapies; NA, not applicable; RoB, risk of bias; Adj., adjusted.

a The *p* values indicate whether the difference between the effect sizes in the subgroups is significant.

b Based on the five main categories of somatic disorders.

Psychotherapy reduced the severity of depression across all main categories of somatic diseases, with variable effects: neurological (*g* = 0.96, 95% CI 0.53–1.38), oncological (*g* = 0.85, 95% CI 0.46–1.25), cardiometabolic (*g* = 0.75, 95% CI 0.51–0.99), HIV/AIDS (*g* = 0.34, 95% CI 0.19–0.50), and other somatic disorders (*g* = 0.48, 95% CI 0.23–0.74). Forest plots for each category of somatic disorders are available in the online Supplementary material (eResults). Subgroup analyses indicated significant differences in effects between categories of somatic disorders (*Q* = 15.17, df = 4, *p* = 0.004). Except for HIV/AIDS (*I*^2^ = 45%), heterogeneity within subgroups of somatic disorders was very high (*I*^2^ > 70%).

Additional subgroup analyses suggested similar effects for all examined moderators (e.g. type of psychotherapy), except for country, with non-Western countries showing significantly larger effects. Additionally, a meta-regression analysis showed that interventions with a higher number of sessions were associated with larger effects (coefficient = 0.04, *p* = 0.038; *R*^2^ = 5.84%).

Sensitivity analyses showed some differences in the estimates when excluding outliers (*g* = 0.55, 95% CI 0.47–0.62; *I^2^* = 28%, 95% CI 1–48), limiting analyses to studies at low RoB (*g* = 0.54, 95% CI 0.33–0.75; *I^2^* = 83%, 95% CI 77–88), and adjusting for publication bias (25 imputed studies, *g* = 0.35, 95% CI 0.18–0.5).

At 6–11 months post-randomization, the effects of psychotherapy on depression severity were *g* = 0.38 (95% CI 0.22–0.53), and *g* = 0.13 (95% CI 0.04–0.21) at 12–24 months post-randomization. Trials employing booster sessions over the follow-ups showed somewhat larger long-term effects (*g* = 0.51, 95% CI 0.20–0.82, *n* = 9) than those without booster sessions (*g* = 0.34, 95% CI 0.14–0.54, *n* = 32), although these differences were not significant (*p* = 0.362). Further analyses on long-term outcomes are available in the Supplementary material (eResults).

### Effects of psychotherapy on quality of life

**Fig. 3.**
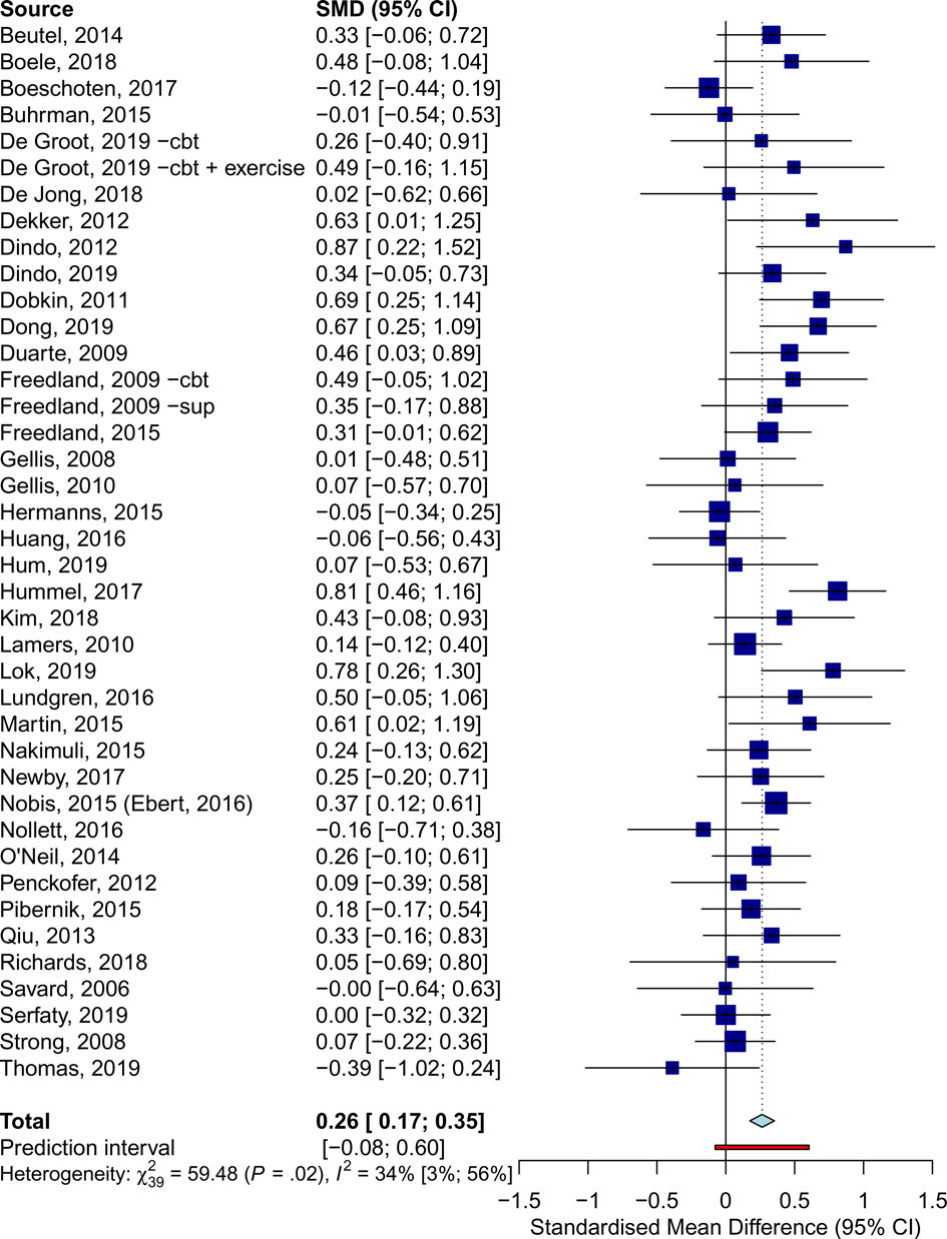
Effects of psychotherapy for depression on overall quality of life across all types of somatic disorders.

Forty comparisons were included in the meta-analysis of *Overall QoL* (Table 2, Fig. 3), estimating a post-treatment effect of *g* = 0.26 (95% CI 0.17–0.35). Effects were *g* = 0.46 (95% CI 0.34–0.57) for *Mental QoL* (eFig. 6), and *g* = 0.22 (95% CI 0.11–0.34) for *Physical QoL* (eFig. 7). Heterogeneity was moderate (*I^2^* = 34%, 95% CI 3–56), and PI were mostly consistent with benefit (−0.08 to 0.60). These results were closely replicated in several sensitivity analyses, and Egger's test indicated no significant publication bias (*p* = 0.369). Additional plots and analyses are presented in the Supplementary material (eResults).

**Table 2.** Effects of psychotherapy on quality of life

	*N*_comp_	*g*	95% CI	*I*^2^	95% CI
Overall QoL	40	0.26	0.17–0.35	34	3–56
Physical QoL	23	0.22	0.11–0.34	32	0–59
Mental QoL	24	0.46	0.34–0.57	36	0–61
Somatic disorders					
Cardiometabolic	17	0.22	0.12–0.32	0	0–50
Oncological	8	0.26	0.09–0.43	27	0–67
Neurological	7	0.43	0.14–0.71	66	23–85
Other	7	0.23	−0.03 to 0.49	61	11–83
Long-term outcomes					
6–11 months	12	0.25	0.16–0.34	0	0–2
⩾12 months	5	0.12	−0.12 to 0.37	24	0–69
Sensitivity analyses					
Outliers excluded	39	0.24	0.16–0.32	23	0–48
Studies at low RoB	23	0.23	0.14–0.33	9	0–43
Adj. for publication bias	46	0.19	0.09–0.29	54	36–67
Only SF-12/SF-36					
Overall	19	0.31	0.22–0.40	0	0–39
PCS	17	0.14	0.04–0.24	0	0–50
MCS	18	0.45	0.31–0.59	47	9–70

*N*_comp_, number of comparisons; *g*, Hedges' *g*; RoB, risk of bias; SF-12, Short-Form 12-item Health Survey Scale; SF-36, Short-Form 36-item Health Survey; PCS, physical component score; MCS, mental component score; Adj., adjusted.

Psychotherapy was effective in improving *Overall QoL* in patients with, neurological (*g* = 0.43, 95% CI 0.14–0.71), oncological (*g* = 0.26, 95% CI 0.09–0.43), and cardiometabolic disorders (*g* = 0.22, 95% CI 0.12–0.32), but no significant effects were observed for other somatic disorders. Subgroup analyses revealed no significant differences between these categories (*Q* = 1.85, df = 3, *p* = 0.604).

At 6–11 months post-randomization, 12 comparisons yielded an effect of *g* = 0.25 (95% CI 0.16–0.34) on *Overall QoL*. Only five studies reported follow-ups longer than 12 months, resulting in a non-significant effect (*g* = 0.12, 95% CI −0.12 to 0.37). No significant differences were detected (*p* = 0.215) between trials that incorporated booster sessions over the follow-up (*g* = 0.29, 95% CI 0.21–0.38, *n* = 6) and those that did not (*g* = 0.20, 95% CI 0.08–0.32, *n* = 14). Further analyses on long-term outcomes are available in the online Supplementary material (eResults).

### Effects of psychotherapy on somatic health-related outcomes

Among a wide range of explored outcomes (e.g. inflammation, blood pressure, cardiac events, lipids, or viral load), only two were reported in a minimum of five studies: glycemic control and pain.

*Glycemic control* (HbA1c) was reported in 10 trials on patients with diabetes and depression (eFig. 8). The pooled estimate indicated no significant effects at post-treatment (*g* = −0.01, 95% CI −0.22 to 0.21; *I*^2^ = 75%, 95% CI 53–87), or at follow-up (*g* = 0.16, 95% CI −0.12 to 0.44), and PI included negative effects (−0.58 to 0.57).

*Pain outcomes* (intensity, severity, or interference) were retrieved from seven trials in chronic pain (*n* = 3), cancer (*n* = 3), and migraine or tension-type headache (*n* = 1). No significant effect was observed at post-treatment (*g* = 0.13, 95% CI −0.21 to 0.47; *I*^2^ = 53%, 95% CI 0–80) (eFig. 9), and PI included negative effects (−0.62 to 0.88).

### Effects of psychotherapy on mortality

All-cause mortality data were reported in 12 studies on oncological (*n* = 4), cardiometabolic (*n* = 3), other somatic disorders (*n* = 4), and HIV (*n* = 1). A total of 50 (4.96%) patients in the psychotherapy groups (*n* = 1009) and 64 (6.31%) patients in the control groups (*n* = 1014) died during the trials. Pooling 11 trials that reported at least one death for one of the conditions, we obtained a non-significant OR = 0.75 (95% CI 0.44–1.29; *I*^2^ = 32%, 95% CI 0–67) (eFig. 10), with PI that included no effects of psychotherapy (PI = 0.44–1.30).

## Discussion

In a systematic review and meta-analysis of 75 RCTs, we observed that psychotherapy reduced the severity of depression across all types of somatic disorders, with very similar effects for different types of psychotherapies, delivery formats, and age groups. Moreover, we observed significant benefits of psychotherapy on the QoL of these patients, both on the mental and physical health-related domains of this outcome. However, we did not find significant effects on any of the examined somatic health-related outcomes (i.e. glycemic control, pain), or mortality.

These findings are in line with earlier meta-analyses, indicating that different types of psychological interventions are effective for depression comorbid to diverse somatic illnesses (Rizzo et al., 2011; van Straten et al., 2010). Meta-analyses focused on specific disorders [e.g. cardiovascular disorders (Reavell, Hopkinson, Clarkesmith, & Lane, 2018), breast cancer (Ye et al., 2018)] also showed similar effects of psychotherapy on depression severity and QoL. The effects of psychotherapy in this population were similar to those observed in depressed adults from the general population, both for depression severity and QoL (Cuijpers, Karyotaki, Reijnders, & Ebert, 2019; Kolovos et al., 2016).

An unexpected finding regarded study drop-out. Participants receiving psychotherapy showed a significantly higher chance of dropping out from the study, compared to control conditions. Further exploratory analyses indicated that drop-out ratios were particularly large in guided self-help interventions, which has been previously observed (Cuijpers, Noma, Karyotaki, Cipriani, & Furukawa, 2019; van Ballegooijen et al., 2014). A potential explanation could be that patients found it hard to combine psychological interventions with complex medical treatments. However, given that in some cases the reasons for drop-out were not available, it is not possible to provide a clear interpretation of this finding. Further research is needed to shed light on the reasons for drop-out.

We observed very large heterogeneity in the effect sizes of depression severity, which was further explored in subgroup analyses. These showed significant differences in effects between Western and non-Western countries, with the latter presenting effects that were twice as large. These differences have been previously observed in psychotherapy research (Cuijpers, Karyotaki, Reijnders, Purgato, & Barbui, 2018), and it could be explained by considerable differences in usual care control conditions across countries (Cuijpers, Quero, Papola, Cristea, & Karyotaki, 2019). In many non-Western countries, usual care could entail not receiving any care. On the other hand, subgroup analyses revealed significant differences between categories of somatic disorders, with HIV/AIDS studies showing the smallest benefits (*g* = 0.34), and neurological disorders the largest (*g* = 0.96). Nevertheless, the differences detected in subgroup analyses did not seem to be the source of heterogeneity, since heterogeneity was still very high even within the subgroups. This uncertainty was also reflected in PI, which were very wide and included negative effects. Trials in HIV/AIDS were the only subgroup of studies with more homogenous effect sizes and with PI mostly consistent with benefit, although showing smaller effects.

A possible reason for these large variations in effects could be related to clinical heterogeneity. Severity and interference of the somatic disorder, type of concomitant medical treatment and its side effects, or prognosis could have an important impact on psychotherapy effects. In this line, two included trials in patients with advanced cancer showed very small benefits of psychotherapy (Lloyd-Williams et al., 2018; Serfaty et al., 2019) whereas trials in cancer patients with much better prognosis (Beutel et al., 2014; Boele et al., 2018) showed much larger effects. This has also been observed with antidepressant pharmacotherapy, where the severity of the comorbid somatic disorders may moderate short- and long-term effects (Reynolds et al., 2006). Conversely, an individual patient data meta-analysis on collaborative care for depression did not find associations between treatment effects and the presence, number, and types of chronic somatic disorders (Panagioti et al., 2016). Personalizing psychotherapy by matching patient profiles to specific types of interventions could be decisive to optimizing treatment effects. However, differences in effects based on patient-level predictors could only be clarified in large RCTs or in individual patient data meta-analyses (Riley, Lambert, & Abo-Zaid, 2010).

The findings of this meta-analysis highlight the importance of addressing mental health symptoms in individuals with medical illnesses. All formats of psychological interventions were effective for not only reducing depressive symptomatology but also enhancing the QoL. Although the effects on QoL were smaller, the clinical significance of this outcome is considerable. Improvements in physical and mental health-related QoL could result in a substantial qualitative impact on the overall functioning of individuals facing the acute and chronic challenges that most somatic illnesses pose.

Our findings suggest that psychological interventions outperform control conditions still 12 months after randomization. However, the effects attenuate over the length of follow-up, which is in line with previous findings (Karyotaki et al., 2016), and some explanations have been suggested. It may be that psychotherapy shows optimal effects during the acute treatment phase and that some patients relapse when this phase is completed. However, it is also possible that this decrease in effects is due to improvements in the control groups, such as treatment seeking or spontaneous remissions (Whiteford et al., 2013). In the context of the included type of patients, another important factor that might have affected long-term mood is the progression of the somatic disorder. The use of booster sessions might be an important resource for enhancing the durability of effects, although the evidence based on the current study is inconclusive.

An additional objective of this study was to assess whether psychotherapy could have an effect on somatic health-related outcomes or mortality. Regarding somatic health-related outcomes, only glycemic control and pain were available, and it was not possible to establish the benefits of psychotherapy on either of them, which is in line with previous research (Cristea, Karyotaki, Hollon, Cuijpers, & Gentili, 2019). Similarly, our results on mortality outcomes are inconclusive. Although there is some meta-analytic evidence suggesting beneficial effects on cardiovascular mortality (Richards et al., 2018), systematic research examining the potential effects of psychotherapy on mortality is scarce. Overall, the few psychotherapy trials reporting mortality or somatic health-related outcomes lacked statistical power and long-term follow-ups, both crucial for drawing conclusions on these outcomes (Katon, 2011; Penninx, Milaneschi, Lamers, & Vogelzangs, 2013).

Further research is needed to obtain a more precise estimation of psychotherapy effects in all examined outcomes, but particularly for mortality and somatic health-related outcomes. This should involve large and high-quality RCTs, including somatic health measures among their outcomes, and following the participants over the long term. Future studies should further investigate the benefit of specific components (e.g. psychoeducation about comorbidity between somatic and mental health problems) for improving outcomes and enhancing adherence to medical treatments. In addition, further examination of combined pharmacotherapy and psychotherapy in this group of patients could provide relevant findings, since combined treatment has been proven effective for depressive symptomatology (Cuijpers et al., 2020) and even for life expectancy (Gallo et al., 2013). Moreover, future research should focus on the long-term maintenance of effects, given the chronic course of most somatic disorders.

### Limitations

The results of this study should be interpreted with caution. First, the quality of the included trials was not optimal, with most of them rated at high RoB. Although challenging to examine, the quality in which psychotherapy is delivered is also an important factor that should be considered. Likewise, publication bias could have led to an overestimation of treatment effects. Moreover, although it is a common finding in psychotherapy (Cuijpers et al., 2020), the high heterogeneity in most of our analyses, which remained unexplained in subgroup analyses, affects the reliability of our findings.

## Conclusions

Although further research is needed to support definitive conclusions, psychotherapy could have a significant clinical impact in patients with depression and somatic disorders. These interventions offer a non-pharmacological alternative to antidepressant medications, which is particularly relevant for patients that are already being treated with multiple pharmacotherapies. Across various modes of delivery, psychological interventions have shown to not only reduce the severity of depression but also to improve quality of life, a crucial outcome for this target group. Considering the chronicity of most somatic disorders, psychotherapy could be a suitable option for pursuing a long-term clinical impact on these patients, by promoting learning, building skills, and facilitating adaptation to living with a physical illness.

## Data Availability

Data and code are available on Open Science Framework (https://osf.io/6cenr/?view_only=7fd3c3ab2e1e48adaacff1c56dfb6522).

## Supplementary material

For supplementary material accompanying this paper visit https://doi.org/10.1017/S0033291721004414. click here to view supplementary material
