# Supplementary material for: Psychotherapy for comorbid depression and somatic disorders: a systematic review and meta-analysis
Source: Psychol Med. 2021 Nov 18;53(6):2503–13. doi: 10.1017/S0033291721004414 (PMC10123840; doi:10.1017/S0033291721004414)

Supplementary material

[eMethods 2](#_Toc61350019)

[Full search string for PubMed 2](#_Toc61350020)

[eTable 1. Overview of outcomes extracted from the included trials 3](#_Toc61350021)

[Algorithm for the selection of depression measures 6](#_Toc61350022)

[eTable 2. Overview of Quality of life instruments 7](#_Toc61350023)

[Changes to protocol 10](#_Toc61350024)

[eResults 11](#_Toc61350025)

[References of included studies 11](#_Toc61350026)

[eTable 3. Characteristics of the 75 included studies 16](#_Toc61350027)

[Effects of psychotherapy on depression severity 20](#_Toc61350028)

[eFigure 1. Cardiometabolic disorders 20](#_Toc61350029)

[eFigure 2. Oncological disorders 20](#_Toc61350030)

[eFigure 3. HIV/AIDS 21](#_Toc61350031)

[eFigure 4. Neurological disorders 21](#_Toc61350032)

[eFigure 5. Other somatic disorders 22](#_Toc61350033)

[Long term outcomes on depression severity 22](#_Toc61350034)

[Effects of psychotherapy on Quality of life 23](#_Toc61350035)

[Additional analyses on Quality of life outcomes 23](#_Toc61350036)

[eFigure 6. Forest plot Mental Quality of life 24](#_Toc61350037)

[eFigure 7. Forest plot Physical Quality of life 24](#_Toc61350038)

[Long term outcomes 25](#_Toc61350039)

[Effects of psychotherapy on somatic health-related outcomes and mortality 26](#_Toc61350040)

[eFigure 8. Forest plot of Glycaemic control 26](#_Toc61350041)

[eFigure 9. Forest plot of Pain outcomes 26](#_Toc61350042)

[eFigure 10. Forest plot of Mortality 27](#_Toc61350043)

# **eMethods**

## **Full search string for PubMed**

(Psychotherapy [MH] OR psychotherap*[All Fields] OR cbt[All Fields] OR "behavior therapies"[All Fields] OR "behavior therapy"[All Fields] OR "behavior therapeutic"[All Fields] OR "behavior therapeutical"[All Fields] OR "behavior therapeutics"[All Fields] OR "behavior therapeutist"[all Fields] OR "behavior therapeutists"[All Fields] OR "behavior treatment"[All Fields] OR "behavior treatments"[All Fields] OR "behaviors therapies"[All Fields] OR "behaviors therapy"[All Fields] OR "behaviors therapeutics"[All Fields] OR "behaviors therapeutic"[All Fields] OR "behaviors therapeutical"[All Fields] OR "behaviors therapeutist"[All Fields] OR "behaviors therapeutists"[All Fields] OR "behaviors treatment"[All Fields] OR "behaviors treatments"[All Fields] OR "behavioral therapies"[All Fields] OR "behavioral therapy"[All Fields] OR "behavioral therapeutics"[All Fields] OR "behavioral therapeutic"[All Fields] OR "behavioral therapeutical"[All Fields] OR "behavioral therapeutist"[All Fields] OR "behavioral therapeutists"[All Fields] OR "behavioral treatment"[All Fields] OR "behavioral treatments"[All Fields] OR "behaviour therapies"[All Fields] OR "behaviour therapy"[All Fields] OR "behaviour therapeutic"[All Fields] OR "behaviour therapeutical"[All Fields] OR "behaviour therapeutics"[All Fields] OR "behaviour therapeutist"[all Fields] OR "behaviour therapeutists"[All Fields] OR "behaviour treatment"[All Fields] OR "behaviour treatments"[All Fields] OR "behaviours therapies"[All Fields] OR "behaviours therapy"[All Fields] OR "behaviours therapeutics"[All Fields] OR "behaviours therapeutic"[All Fields] OR "behaviours therapeutical"[All Fields] OR "behaviours therapeutist"[All Fields] OR "behaviours therapeutists"[All Fields] OR "behaviours treatment"[All Fields] OR "behaviours treatments"[All Fields] OR "behavioural therapies"[All Fields] OR "behavioural therapy"[All Fields] OR "behavioural therapeutics"[All Fields] OR "behavioural therapeutic"[All Fields] OR "behavioural therapeutical"[All Fields] OR "behavioural therapeutist"[All Fields] OR "behavioural therapeutists"[All Fields] OR "behavioural treatment"[All Fields] OR "behavioural treatments"[All Fields] OR "cognition therapies"[All Fields] OR "cognition therapie"[All Fields] OR "cognition therapy"[All Fields] OR "cognition therapeutical"[All Fields] OR "cognition therapeutic"[All Fields] OR "cognition therapeutics"[All Fields] OR "cognition therapeutist"[All Fields] OR "cognition therapeutists"[All Fields] OR "cognition treatment"[All Fields] OR "cognition treatments"[All Fields] OR psychodynamic[All Fields] OR Psychoanalysis[MH] OR psychoanalysis[All Fields] OR psychoanalytic*[All Fields] OR counselling[All Fields] OR counseling[All Fields] OR Counseling[MH] OR "problem-solving"[All Fields] OR mindfulness[All Fields] OR (acceptance[All Fields] AND commitment[All Fields] ) OR "assertiveness training"[All Fields] OR "behavior activation"[All Fields] OR "behaviors activation"[All Fields] OR "behavioral activation"[All Fields] OR "cognitive therapies"[All Fields] OR "cognitive therapy"[All Fields] OR "cognitive therapeutic"[All Fields] OR "cognitive therapeutics"[All Fields] OR "cognitive therapeutical"[All Fields] OR "cognitive therapeutist"[All Fields] OR "cognitive therapeutists"[All Fields] OR "cognitive treatment"[All Fields] OR "cognitive treatments"[All Fields] OR "cognitive restructuring"[All Fields] OR (("compassion-focused"[All Fields] OR "compassion-focussed"[All Fields]) AND (therapy[SH] OR therapies[All Fields] OR therapy[All Fields] OR therape*[All Fields] OR therapis*[All Fields]OR Therapeutics [OR treatment*[All Fields])) OR ((therapy[SH] OR therapies[All Fields] OR therapy [All Fields] OR therape*[All Fields] OR therapis*[All Fields] OR Therapeutics[MH] OR treatment*[All Fields]) AND constructivist*[All Fields]) OR "metacognitive therapies"[All Fields] OR "metacognitive therapy"[All Fields] OR "metacognitive therapeutic"[All Fields] OR "metacognitive therapeutics"[All Fields] OR "metacognitive therapeutical"[All Fields] OR "metacognitive therapeutist"[All Fields] OR "metacognitive therapeutists"[All Fields] OR "metacognitive treatment"[All Fields] OR "metacognitive treatments"[All Fields] OR "meta-cognitive therapies"[All Fields] OR "meta-cognitive therapy"[All Fields] OR "meta-cognitive therapeutic"[All Fields] OR "meta-cognitive therapeutics"[All Fields] OR "meta-cognitive therapeutical"[All Fields] OR "meta-cognitive therapeutist"[All Fields] OR "meta-cognitive therapeutists"[All Fields] OR "meta-cognitive treatment"[All Fields] OR "meta-cognitive treatments"[All Fields] OR "solution-focused therapies"[All Fields] OR "solution-focused therapy"[All Fields] OR "solution-focused therapeutic"[All Fields] OR "solution-focused therapeutics"[All Fields] OR "solution-focused therapeutical"[All Fields] OR "solution focused therapies"[All Fields] OR "solution focused therapy"[All Fields] OR "solution focused therapeutic"[All Fields] OR "solution focused therapeutics"[All Fields] OR "solution focused therapeutical"[All Fields]OR "solution-focussed therapies"[All Fields] OR "solution-focussed therapy"[All Fields] OR "solution-focussed therapeutic"[All Fields] OR "solution-focussed therapeutics"[All Fields] OR "solution-focussed therapeutical"[All Fields]OR "solution focussed therapies"[All Fields] OR "solution focussed therapy"[All Fields] OR "solution focussed therapeutic"[All Fields] OR "solution focussed therapeutics"[All Fields] OR "solution focussed therapeutical"[All Fields] OR "self-control therapies"[All Fields] OR "self-control therapy"[All Fields] OR "self-control therapeutics"[All Fields] OR "self-control therapeutical"[All Fields] OR "self-control therapeutic"[All Fields] OR "self-control training"[All Fields] OR "self-control trainings"[All Fields] OR "self control therapies"[All Fields] OR "self control therapy"[All Fields] OR "self control therapeutics"[All Fields] OR "self control therapeutical"[All Fields] OR "self control therapeutic"[All Fields] OR "self control training"[All Fields] OR "self control trainings"[All Fields] AND (Depressive Disorder[MH] OR Depression[MH]OR dysthymi*[All Fields] OR "affective disorder"[All Fields]OR "affective disorders"[All Fields] OR "mood disorder"[All Fields] OR "mood disorders"[All Fields] OR depression*[All Fields] OR depressive*[All Fields] OR "dysthymic disorder"[MeSH Terms]) AND ((randomized controlled trial [pt] OR controlled clinical trial [pt] OR randomized [tiab] OR randomly [tiab] NOT (animals[mh] NOT (animals[mh] AND humans [mh]))

## **eTable 1. Overview of outcomes extracted from the included trials**

| **Study** | **Category of somatic disorder** | **Depression** | **Quality of life** | **Glycemic index** | **Pain** | **Mortality** |
| --- | --- | --- | --- | --- | --- | --- |
| Abas, 2018 | HIV | PHQ-9 | - | - | - | - |
| Ahmadpanah, 2016 | Cardiometabolic | BDI | - | - | - | - |
| Bedard, 2014 | Other | BDI-II | - | - | - | - |
| Beutel, 2014 | Oncological | HADS-D | EORTC-QLQ-C30 | - | EORTC-QLQ-C30 - Pain | - |
| Boele, 2018 | Oncological | CES-D | SF-36 | - | - | - |
| Boeschoten, 2017 | Neurological | BDI-II | EQ-5D | - | - | - |
| Buhrman, 2015 | Other | MADRS-S | QOLI | - | MPI - Pain severity | - |
| Burns, 2007 | Other | PHQ-9 | - | - | - | - |
| Chesney, 2003 | HIV | CES-D | - | - | - | - |
| De Groot, 2019 | Cardiometabolic | BDI-II | SF-12 | HbA1c | - | - |
| De Jong, 2018 | Other | HAM-D | SF-36 | - | SF-36 - Bodily pain | - |
| Dekker, 2012 | Cardiometabolic | BDI-II | MLHF | - | - | - |
| Desautels, 2017 | Oncological | HAM-D | - | - | - | - |
| Dindo, 2012 | Neurological | HAM-D | SF-36 | - | - | - |
| Dindo, 2019 | Neurological | HAM-D | WHOQOL-BREF | - | - | - |
| Dobkin, 2011 | Neurological | HAM-D | SF-36 | - | - | - |
| Doering, 2013 | Cardiometabolic | BDI | - | - | - | - |
| Dong, 2019 | Oncological | HAM-D | MUNSH | - | - | - |
| Duarte, 2009 | Other | BDI | SF-36 | - | - | CONSORT flow diagram |
| Evans, 1995 | Oncological | CES-D | - | - | - | - |
| Fann, 2015 | Other | HAM-D | - | - | - | CONSORT flow diagram |
| Freedland, 2009 | Cardiometabolic | HAM-D | SF-36 | - | - | - |
| Freedland, 2015 | Cardiometabolic | HAM-D | SF-12 | - | - | - |
| Gellis, 2008 | Cardiometabolic | HAM-D | QOLI | - | - | - |
| Gellis, 2010 | Other | HAM-D | SF-36 | - | - | - |
| Heckman, 2011 | HIV | GDS | - | - | - | - |
| Heckman, 2013 | HIV | GDS | - | - | - | - |
| Heckman, 2017 | HIV | BDI | - | - | - | - |
| Hermanns, 2015 | Cardiometabolic | PHQ-9 | EQ-5D | HbA1c | - | - |
| Herrmann-Lingen, 2016 | Cardiometabolic | HADS-D | - | - | - | Mortality reported as part of safety analysis |
| Huang, 2016 | Cardiometabolic | CES-D | SF-36 | HbA1c | - | - |
| Hum, 2019 | Neurological | QIDS-SR | WHOQOL-BREF | - | - | - |
| Hummel, 2017 | Other | HAM-D | IADL | - | - | Mortality reported among trial outcomes |
| Jalali, 2019 | HIV | BDI-II | - | - | - | - |
| Kamga, 2017 | Other | PHQ-9 | - | - | - | - |
| Kelly, 1993 | HIV | CES-D | - | - | - | - |
| Kim, 2018 | Oncological | HADS-D | EORTC-QLQ-C30 | - | EORTC-QLQ-C30 - Pain | - |
| Lamers, 2010 | Other | BDI | SF-36 | - | - | CONSORT flow diagram |
| Larcombe, 1984 | Neurological | HAM-D | - | - | - | - |
| Lloyd-Williams, 2018 | Oncological | PHQ-9 | - | - | - | Survival reported as trial outcome |
| Lök, 2019 | Neurological | CSDD | QOL-AD | - | - | - |
| Lundgren, 2016 | Cardiometabolic | PHQ-9 | MLHF | - | - | - |
| Lustman, 1998 | Cardiometabolic | BDI | - | GHb | - | - |
| Martin, 2015 | Neurological | BDI-II | SF-36 | - | SF-36 - Bodily pain | - |
| Mohr, 2000 | Neurological | POMS-D | - | - | - | - |
| Mossey, 1996 | Other | GDS | - | - | - | - |
| Nakimuli, 2015 | HIV | SRQ-20 | Function assessment (locally developed) | - | - | CONSORT flow diagram |
| Newby, 2017 | Cardiometabolic | PHQ-9 | SF-12 | HbA1c | - | - |
| Nobis, 2015 | Cardiometabolic | CES-D | SF-12 | HbA1c | - | - |
| Nollett, 2016 | Other | BDI-II | EQ-5D | - | - | - |
| Olukolade, 2017 | Cardiometabolic | BDI | - | - | - | - |
| O'Neil, 2014 | Cardiometabolic | PHQ-9 | SF-12 | - | - | - |
| Onuigbo, 2019 | Other | BDI-II | - | - | - | - |
| Penckofer, 2012 | Cardiometabolic | CES-D | SF-12 | HbA1c | - | - |
| Petersen, 2014 | HIV | PHQ-9 | - | - | - | - |
| Pibernik, 2015 | Cardiometabolic | CES-D | SF-12 | HbA1c | - | - |
| Poleshuck, 2014 | Other | HAM-D | - | - | MPI- Pain severity | - |
| Qiu, 2013 | Oncological | HAM-D | FACT-B | - | - | - |
| Ransom, 2008 | HIV | BDI-II | - | - | - | - |
| Richards, 2018 | Cardiometabolic | BDI-II | EQ-5D | - | - | “No deaths were recorded in either arm of the trial between baseline and the final follow-up” |
| Safren, 2009 | HIV | HAM-D | - | - | - | - |
| Safren, 2014 | Cardiometabolic | MADRS | - | HbA1c | - | - |
| Safren, 2016 | HIV | MADRS | - | - | - | - |
| Savard, 2006 | Oncological | HAM-D | EORTC-QLQ-C30 | - | - | CONSORT flow diagram |
| Serfaty, 2019 | Oncological | BDI-II | EQ-5D | - | - | CONSORT flow diagram |
| Simoni, 2013 | HIV | BDI | - | - | - | - |
| Simson, 2008 | Cardiometabolic | HADS-D | - | - | - | - |
| Strong, 2008 | Oncological | SCL20-D | EORTC-QLQ-C30 | - | EORTC-QLQ-C30 - Pain | CONSORT flow diagram |
| Taylor, 2009 | Cardiometabolic | HAM-D | - | - | - | - |
| Teri, 1997 | Neurological | HAM-D | - | - | - | - |
| Thomas, 2019 | Cardiometabolic | PHQ-9 | EQ-5D | - | - | - |
| Tovote, 2014 | Cardiometabolic | HAM-D7 | - | - | - | - |
| Turner, 2013 | Cardiometabolic | BDI-II | - | - | - | CONSORT flow diagram |
| Van Bastelaar, 2011 | Cardiometabolic | CES-D | - | HbA1c | - | - |
| Zhao, 2019 | Other | EPDS | - | - | - | - |

Abbreviations:

*Depression*

BDI (Beck Depression Inventory); PHQ-9 (Patient Health Questionnaire); HAM-D (Hamilton Depression Rating Scale); HADS-D (Hospital Anxiety and Depression Scale–Depression subscale); MADRS (Montgomery–Åsberg Depression Rating Scale); CES-D (Center for Epidemiological Studies Depression Scale); GDS (Geriatric Depression Scale); QIDS-SR (Quick Inventory of Depressive Symptomatology–Self-report); CSDD (Cornell Scale for Depression in Dementia); POMS-D (Profile of Mood States – Depression); SRQ-20 (Self Reporting Questionnaire); SCL (Hopkins Symptom Checklist-20); EPDS (Edinburgh Postnatal Depression Scale)

*Quality of life*

EORTC-QLQ-C30 (European Organisation for Research and Treatment of Cancer Quality of Life questionnaire); EQ-5D-5 (European Quality of Life – 5 Dimensions); FACT (Functional assessment of Cancer Therapy – Breast); IADL (Instrumental Activities of Daily Living); MLHF (Minnesota Living with Heart Failure Questionnaire); MUNSH (Memorial University of Newfoundland Scale of Happiness); QOL‐AD (Quality of Life in Alzheimer's Disease); QOLI (Quality of Life Inventory); SF-12 (Short-Form 12 Health Survey Scale); SF-36 (Short-Form 36-item Health Survey); WHOQOL-BREF (World Health Organization Quality of Life- BREF)

*Glycemic index*

GHb (glycosylated hemoglobin); HbA1c (glycated hemoglobin)

*Pain*

[MPI (Multidimensional Pain Inventory)](http://scireproject.com/outcome-measures/outcome-measure-tool/multidimensional-pain-inventory-mpi-sci-version/)

## **Algorithm for the selection of depression measures**

We selected the depression measures in the following order:

- HAMD
- BDI I or II
- Another clinician-rated instrument;
- Another self-report instrument, with priority for:
  - PHQ-9
  - CES-D
  - HADS-D
  - GDS over IDS
  - HADSD over POMS
  - EPDS over DASS-D
  - MMPI-D over DACL
  - Zung over Lubin

**Development of the algorithm:**

The algorithm was built by prioritizing the HAM-D, due to being clinician-rated (thus allowing blinding), and due to being a widely used instrument in depression therapy research, both in psychotherapy and pharmacotherapy. The next prioritized instrument was the BDI, given that is the most frequently used instrument in our database. After the BDI, we prioritized any clinician-rated instrument over self-reports. If only self-reports were reported, we selected one following the above order, which is based on their frequency of use and psychometric properties.

## **eTable 2. Overview of Quality of life instruments**

| **Instrument** | **Description** | **Subscales** | **Trials in which is included** |
| --- | --- | --- | --- |
| EORTC-QLQ-C30  (European Organisation for Research and Treatment of Cancer (EORTC) Quality of Life questionnaire) | Self-report assessing cancer patients’ quality of life. It contains 15 subscales within 3 domains that measure global health status/quality of life, functional status and symptom status. | - Global quality of life:  1. Global health status  - Functional status:  1. physical function 2. role function 3. emotional function 4. cognitive function 5. social function  - Symptom status:  1. Fatigue 2. nausea and vomiting 3. pain 4. dyspnoea 5. insomnia 6. appetite loss 7. constipation 8. diarrhea 9. financial difficulties | Beutel, 2014  Kim, 2018  Savard, 2006  Strong, 2008 |
| EQ-5D-5  (European Quality of Life – 5 Dimensions) | Standardized self-report measure of HRQL that provides a generic measure of health for clinical and economic appraisal. It consist of two main parts: 1) descriptive system using a 5-dimension scale, resulting in an index score; 2) visual analogue scale (VAS), resulting in a total score (0-100). | - Index:  1. mobility 2. self-care 3. usual activities 4. pain/discomfort 5. anxiety/depression  - VAS | Hermanns, 2015  Richards, 2018  Boeschoten, 2017  Nollett, 2016  Serfaty, 2019  Thomas, 2019 |
| FACT-B  (Functional assessment of Cancer Therapy – Breast) | 44-item self-report instrument designed to measure multidimensional quality of life (QL) in patients with breast cancer. | - Physical Well-Being (PWB) - Emotional Well-Being (EWB) - Social Well-Being (SWB) - Functional Well-Being (FWB) - Relationship with Doctor (RWD). | Qiu, 2013 |
| Function assessment method locally developed (in Nakimuli et al., 2015) | 5-items scale. Items were derived from qualitative interviews with individuals and their caregivers. These interviews were about their expectations regarding function outcomes. | 1. household (e.g. washing clothes, sweeping the yard) 2. field (digging, grazing animals) 3. social (attending social events) 4. job or school-related tasks (participating in income-generating activities, attending school or skills training courses) 5. Tasks related to personal hygiene (eg, bathing). | Nakimuli, 2015 |
| IADL  (Instrumental Activities of Daily Living) | Scale assessing everyday functional competence | Ability to use telephone, shopping, food preparation, housekeeping, laundry, mode of transportation, responsibility for own medication, ability to handle finances… | Hummel, 2017 |
| MLHF  (Minnesota Living with Heart Failure Questionnaire) | Self-report measuring health-related quality of life (HRQL), including 21 items assessing physical, emotional and socioeconomic ways in which heart failure can adversely affect a patient’s life. | 1. Physical 2. Socio-economic 3. Emotional/psychological aspects | Dekker, 2012  Lundgren, 2016 |
| MUNSH  (Memorial University of Newfoundland Scale of Happiness) | Self-report instrument of subjective well-being with 24 items that address affect during the preceding month and longer-term affective experiences. | Four components:   1. Positive Affect 2. Negative Affect 3. Positive Experience 4. Negative Experience | Dong, 2019 |
| QOL‐AD  (Quality of Life in Alzheimer's Disease) | Scale of 13 items providing information about quality of life in patients with Alzheimer Disease. This scale assesses a variety of life domains, including the patient's physical health, mood, relationships, activities, and ability to complete tasks. | Scoring is based on the simple aggregation of the points obtained from all items. | Lök, 2019 |
| QOLI  (Quality of Life Inventory) | 32 items assessing multidimensional quality of life and life satisfaction. | The assessment yields an overall score and profile in 16 areas of life; health, self-esteem, goals and values, money, work, play, learning, creativity, helping, love, friends, children, relatives, home, neighborhood, and community. | Buhrman, 2015  Gellis, 2008 |
| SF-12  (Short-Form 12-item Health Survey Scale) | 12-item short form self-report derived from the SF-36. It consists of 8 domains measuring HRQL, and scores can be summarized into a mental component score (MCS) and a physical component score (PCS). | - PCS:  1. General health 2. Physical function 3. Role physical 4. Bodily pain  - MCS:  1. Mental health 2. Role emotional 3. Social function 4. Vitality | Freedland, 2015  Newby, 2017  Nobis, 2015 (Ebert, 2016)  O'Neil, 2014  Penckofer, 2012  Pibernik, 2015  De Groot, 2019 |
| SF-36  (Short-Form 36-item Health Survey) | 36-item, patient-reported survey of patient health. It consists of 8 domains measuring HRQL, and scores can be summarized into a mental component score (MCS) and a physical component score (PCS). | - PCS:  1. General health 2. Physical function 3. Role physical 4. Bodily pain  - MCS:  1. Mental health 2. Role emotional 3. Social function 4. Vitality | Freedland, 2009  Gellis, 2010  Huang, 2016  Dindo, 2012  Dobkin, 2011  Martin, 2015  Boele, 2018  De Jong, 2018  Duarte, 2009  Lamers, 2010 |
| WHOQOL-BREF  (World Health Organization Quality of Life- BREF) | 26-item questionnaire that assesses quality of life across four domains: physical health, psychological health, social relationships, and the environment. | Domains:   1. Psychological health 2. Social relationships 3. Environment 4. Physical health | Dindo, 2019  Hum, 2019 |

## **Changes to** **protocol**

In the registered protocol for this systematic review and meta-analysis (<https://osf.io/q6z3p>) we specified that the main outcomes of this study would be “depression, quality of life, functioning, and health-related outcomes”. Given the substantial overlap in the concepts of quality of life and functioning we decided to include only quality of life as an overarching outcome, which in some occasions included measures described as “functioning”.

# **eResults**

## **References of included studies**

| Study | Full reference |
| --- | --- |
| Abas, 2018 | Abas M, Nyamayaro P, Bere T, et al. Feasibility and acceptability of a task-shifted intervention to enhance adherence to HIV medication and improve depression in people living with HIV in Zimbabwe, a low income country in sub-Saharan Africa. AIDS and Behavior 2018; 22(1): 86-101. |
| Ahmadpanah, 2016 | Ahmadpanah, M., Paghale, S. J., Bakhtyari, A., Kaikhavani, S., Aghaei, E., Nazaribadie, M., . . . Brand, S. (2016). Effects of psychotherapy in combination with pharmacotherapy, when compared to pharmacotherapy only on blood pressure, depression, and anxiety in female patients with hypertension. |
| Bedard, 2014 | Bedard M, Felteau M, Marshall S, Cullen N, Gibbons C, Dubois S, et al. Mindfulness-based cognitive therapy reduces symptoms of depression in people with a traumatic brain injury: Results from a randomized controlled trial. Journal of Head Trauma Rehabilitation. 2014;29(4):E13-E22. |
| Beutel, 2014 | Beutel ME, Weissflog G, Leuteritz K, Wiltink J, Haselbacher A, Ruckes C, et al. Efficacy of short-term psychodynamic psychotherapy (STPP) with depressed breast cancer patients: Results of a randomized controlled multicenter trial. Annals of Oncology. 2014;25(2):378-84. |
| Boele, 2018 | Boele FW, Klein M, Verdonck-de Leeuw IM, et al. Internet-based guided self-help for glioma patients with depressive symptoms: a randomized controlled trial. Journal of neuro-oncology 2018; 137(1): 191‐203. |
| Boeschoten, 2017 | Boeschoten, R. E., Dekker, J., Uitdehaag, B. M. J., Beekman, A. T. F., Hoogendoorn, A. W., Collette, E. H., . . . Van Oppen, P. (2017). Internet-based treatment for depression in multiple sclerosis: A randomized controlled trial. Multiple Sclerosis, 23(8), 1112-1122. |
| Buhrman, 2015 | Buhrman M, Syk M, Burvall O, Hartig T, Gordh T, Andersson G. Individualized Guided Internet-delivered Cognitive Behaviour Therapy for Chronic Pain Patients with Comorbid Depression and Anxiety: A Randomized Controlled Trial. Clinical Journal of Pain 2014. |
| Burns, 2007 | Burns A, Banerjee S, Morris J, Woodward Y, Baldwin R, Proctor R, et al. Treatment and prevention of depression after surgery for hip fracture in older people: Randomized, controlled trials. Journal of the American Geriatrics Society. 2007;55(1):75-80. |
| Chesney, 2003 | Chesney MA, Chambers DB, Taylor JM, Johnson LM, Folkman S. Coping effectiveness training for men living with HIV: Results from a randomized clinical trial testing a group-based intervention. Psychosomatic Medicine. 2003;65(6):1038-46. |
| De Groot, 2019 | De Groot M, Shubrook JH, Hornsby WG, Pillay Y, Mather KJ, Fitzpatrick K, . . . Saha C. Program ACTIVE II: Outcomes from a randomized, multistate community-based depression treatment for rural and urban adults with type 2 diabetes. Diabetes Care. 2019; 42(7), 1185-1193. |
| De Jong, 2018 | De Jong M, Peeters F, Gard T, et al. A randomized controlled pilot study on mindfulness-based cognitive therapy for unipolar depression in patients with chronic pain. Journal of clinical psychiatry 2018; 79(1): 26‐34. |
| Dekker, 2012 | Dekker RL, Moser DK, Peden AR, Lennie TA. Cognitive therapy improves three-month outcomes in hospitalized patients with heart failure. Journal of cardiac failure. 2012;18(1):10-20. |
| Desautels, 2017 | Desautels C, Savard J, Ivers H, Savard MH, Caplette-Gingras, A. Treatment of Depressive Symptoms in Patients with Breast Cancer: A Randomized Controlled Trial Comparing Cognitive Therapy and Bright Light Therapy. Health Psychology. 2017; 37(1), 1. |
| Dindo, 2012 | Dindo L, Recober A, Marchman JN, Turvey C, O'Hara MW. One-day behavioral treatment for patients with comorbid depression and migraine: A pilot study. Behaviour Research and Therapy. 2012;50(9):537-43. |
| Dindo, 2019 | Dindo LN, Recober A, Calarge CA, Zimmerman BM, Weinrib A, Marchman JN, Turvey C. One-Day Acceptance and Commitment Therapy Compared to Support for Depressed Migraine Patients: a Randomized Clinical Trial. Neurotherapeutics. 2019. |
| Dobkin, 2011 | Dobkin RD, Menza M, Allen LA, Gara MA, Mark MH, Tiu J, et al. Cognitive-behavioral therapy for depression in Parkinson's disease: A randomized, controlled trial. American Journal of Psychiatry. 2011;168(10):1066-74. |
| Doering, 2013 | Doering LV, Chen B, Cross Bodan R, Magsarili MC, Nyamathi A, Irwin MR. Early cognitive behavioral therapy for depression after cardiac surgery. Journal of Cardiovascular Nursing. 2013;28(4):370-9. |
| Dong, 2019 | Dong X, Sun G, Zhan J, Liu F, Ma S, Li P, . . . Liu Y. Telephone-based reminiscence therapy for colorectal cancer patients undergoing postoperative chemotherapy complicated with depression: a three-arm randomised controlled trial. Supportive care in cancer: official journal of the Multinational Association of Supportive Care in Cancer. 2019; 27(8), 2761-2769. |
| Duarte, 2009 | Duarte PS, Miyazaki MC, Blay SL, Sesso R. Cognitive-behavioral group therapy is an effective treatment for major depression in hemodialysis patients. Kidney international. 2009;76(4):414-21. |
| Evans, 1995 | Evans RL, Connis RT. Comparison of brief group therapies for depressed cancer patients receiving radiation treatment. Public Health Reports. 1995;110(3):306-11. |
| Fann, 2015 | Fann JR, Bombardier CH, Vannoy S, Dyer J, Ludman E, Dikmen S, et al. Telephone and in-person cognitive behavioral therapy for major depression after traumatic brain injury: A randomized controlled trial. Journal of neurotrauma. 2015;32(1):45-57. |
| Freedland, 2009 | Freedland KE, Skala JA, Carney RM, Rubin EH, Lustman PJ, D·vila-Rom·n VG, et al. Treatment of depression after coronary artery bypass surgery: A randomized controlled trial. Archives of General Psychiatry. 2009;66(4):387-96. |
| Freedland, 2015 | Freedland KE, Carney RM, Rich MW, Steinmeyer BC, Rubin EH. Cognitive behavior therapy for depression and self-care in heart failure patients: A randomized clinical trial. JAMA Internal Medicine. 2015;175(11):1773-82. |
| Gellis, 2008 | Gellis ZD, McGinty J, Tierney L, Jordan C, Burton J, Misener E. Randomized controlled trial of problem-solving therapy for minor depression in home care. Research on Social Work Practice. 2008;18(6):596-606. |
| Gellis, 2010 | Gellis ZD, Bruce ML. Problem solving therapy for subthreshold depression in home healthcare patients with cardiovascular disease. The American Journal of Geriatric Psychiatry. 2010;18(6):464-74. |
| Heckman, 2011 | Heckman TG, Sikkema KJ, Hansen N, Kochman A, Heh V, Neufeld S, et al. A randomized clinical trial of a coping improvement group intervention for HIV-infected older adults. Journal of behavioral medicine. 2011;34(2):102-11. |
| Heckman, 2013 | Heckman TG, Heckman BD, Anderson T, Lovejoy TI, Mohr D, Sutton M, . . . Gau JT. Supportive-expressive and coping group teletherapies for HIV-infected older adults: a randomized clinical trial. AIDS Behav. 2013;17(9), 3034-3044. |
| Heckman, 2017 | Heckman, T. G., Heckman, B. D., Anderson, T., Lovejoy, T. I., Markowitz, J. C., Shen, Y., & Sutton, M. (2017). Tele-interpersonal psychotherapy acutely reduces depressive symptoms in depressed HIV-infected rural persons: A randomized clinical trial. Behavioral Medicine, 43(4), 285-295. |
| Hermanns, 2015 | Hermanns N, Schmitt A, Gahr A, Herder C, Nowotny B, Roden M, et al. The effect of a diabetes-specific cognitive behavioral treatment program (DIAMOS) for patients with diabetes and subclinical depression: Results of a randomized controlled trial. Diabetes care. 2015;38(4):551-60. |
| Herrmann-Lingen, 2016 | Herrmann-Lingen C, Beutel ME, Bosbach A, Deter HC, Fritzsche K, Hellmich M, et al. A stepwise psychotherapy intervention for reducing risk in coronary artery disease (SPIRR-CAD): Results of an observer-blinded, multicenter, randomized trial in depressed patients with coronary artery disease. Psychosomatic Medicine. 2016;78(6):704-15. |
| Huang, 2016 | Huang CY, Lai HL, Chen CI, Lu YC, Li SC, Wang LW, Su Y. Effects of motivational enhancement therapy plus cognitive behaviour therapy on depressive symptoms and health-related quality of life in adults with type II diabetes mellitus: A randomised controlled trial. Quality of Life Research: An International Journal of Quality of Life Aspects of Treatment, Care & Rehabilitation. 2016; 25(5), 1275-1283. |
| Hum, 2019 | Hum KM, Chan CJ, Gane J, Conway L, McAndrews MP, Smith ML. Do distance-delivery group interventions improve depression in people with epilepsy?. Epilepsy & Behavior. 2019; 98, 153-160. |
| Hummel, 2017 | Hummel, J., Weisbrod, C., Boesch, L., Himpler, K., Hauer, K., Hautzinger, M., . . . Kopf, D. (2017). AIDE–Acute Illness and Depression in Elderly Patients. Cognitive Behavioral Group Psychotherapy in Geriatric Patients With Comorbid Depression: A Randomized, Controlled Trial. Journal of the american medical directors association, 18(4), 341-349. |
| Jalali, 2019 | Jalali F, Hasani A, Hashemi SF, Kimiaei SA, Babaei A. Cognitive Group Therapy Based on Schema-Focused Approach for Reducing Depression in Prisoners Living With HIV. International journal of offender therapy and comparative criminology. 2019; 63(2), 276‐288. |
| Kamga, 2017 | Kamga, H., McCusker, J., Yaffe, M., Sewitch, M., Sussman, T., Strumpf, E., . . . Freeman, E. (2017). Self-care tools to treat depressive symptoms in patients with age-related eye disease: a randomized controlled clinical trial. Clinical & experimental ophthalmology, 45(4), 371-378. |
| Kelly, 1993 | Kelly JA, Murphy DA, Bahr GR, Kalichman SC, Morgan MG, Stevenson LY, et al. Outcome of cognitive-behavioral and support group brief therapies for depressed, HIV-infected persons. American Journal of Psychiatry. 1993;150(11):1679-86. |
| Kim, 2018 | Kim YH, Choi KS, Han K, Kim HW. A psychological intervention programme for patients with breast cancer under chemotherapy and at a high risk of depression: a randomised clinical trial. Journal of clinical nursing 2018; 27(3‐4): 572‐81. |
| Lamers, 2010 | Lamers F, Jonkers CC, Bosma H, Kempen GI, Meijer JA, Penninx BW, et al. A minimal psychological intervention in chronically ill elderly patients with depression: A randomized trial. Psychotherapy and Psychosomatics. 2010;79(4):217-26. |
| Larcombe, 1984 | Larcombe NA, Wilson PH. An evaluation of cognitive-behaviour therapy for depression in patients with multiple sclerosis. The British Journal of Psychiatry. 1984;145:366-71. |
| Lloyd-Williams, 2018 | Lloyd-Williams, M., Shiels, C., Ellis, J., Abba, K., Gaynor, E., Wilson, K., & Dowrick, C. (2018). Pilot randomised controlled trial of focused narrative intervention for moderate to severe depression in palliative care patients: DISCERN trial. Palliative medicine, 32(1), 206‐215. |
| Lök, 2019 | Lök, N., Bademli, K., & Selçuk-Tosun, A. (2019). The effect of reminiscence therapy on cognitive functions, depression, and quality of life in Alzheimer patients: randomized controlled trial. International journal of geriatric psychiatry, 34(1), 47‐53. |
| Lundgren, 2016 | Lundgren JG, Dahlstrom O, Andersson G, Jaarsma T, Karner Kohler A, Johansson P. The effect of guided web-based cognitive behavioral therapy on patients with depressive symptoms and heart failure: A pilot randomized controlled trial. Journal of Medical Internet Research. 2016;18(8):e194. |
| Lustman, 1998 | Lustman PJ, Griffith LS, Freedland KE, Kissel SS, Clouse RE. Cognitive behavior therapy for depression in type 2 diabetes mellitus. A randomized, controlled trial. Annals of Internal Medicine. 1998;129(8):613-21. |
| Martin, 2015 | Martin PR, Aiello R, Gilson K, Meadows G, Milgrom J, Reece J. Cognitive behavior therapy for comorbid migraine and/or tension-type headache and major depressive disorder: An exploratory randomized controlled trial. Behaviour Research and Therapy. 2015;73:8-18. |
| Mohr, 2000 | Mohr DC, Likosky W, Bertagnolli A, Goodkin DE, Van Der Wende J, Dwyer P, et al. Telephone-administered cognitive-behavioral therapy for the treatment of depressive symptoms in multiple sclerosis. Journal of Consulting and Clinical Psychology. 2000;68(2):356-61. |
| Mossey, 1996 | Mossey JM, Knott KA, Higgins M, Talerico K. Effectiveness of a psychosocial intervention, interpersonal counseling, for subdysthymic depression in medically ill elderly. The journals of gerontology Series A, Biological sciences and medical sciences. 1996;51(4):M172-8. |
| Nakimuli, 2015 | Nakimuli-Mpungu E, Wamala K, Okello J, Alderman S, Odokonyero R, Mojtabai R, et al. Group support psychotherapy for depression treatment in people with HIV/AIDS in northern Uganda: A single-centre randomised controlled trial. The Lancet HIV. 2015;2(5):e190-e9. |
| Newby, 2017 | Newby, J., Robins, L., Wilhelm, K., Smith, J., Fletcher, T., Gillis, I., . . . Andrews, G. (2017). Web-Based Cognitive Behavior Therapy for Depression in People With Diabetes Mellitus: a Randomized Controlled Trial. Journal of medical Internet research, 19(5), e157. |
| Nobis, 2015 | Nobis S, Lehr D, Ebert DD, Baumeister H, Snoek F, Riper H, et al. Efficacy of a web-based intervention with mobile phone support in treating depressive symptoms in adults with type 1 and type 2 diabetes: A randomized controlled trial. Diabetes care. 2015;38(5):776-83. |
| Nollett, 2016 | Nollett CL, Bray N, Bunce C, Casten RJ, Edwards RT, Hegel MT, et al. Depression in Visual Impairment Trial (DEPVIT): A randomized clinical trial of depression treatments in people with low vision. Investigative Ophthalmology and Visual Science. 2016;57(10):4247-54. |
| Olukolade, 2017 | Olukolade, O., & Osinowo, H. (2017). Efficacy of Cognitive Rehabilitation Therapy on Poststroke Depression among Survivors of First Stroke Attack in Ibadan, Nigeria. Behavioural Neurology, 2017. |
| O'Neil, 2014 | O'Neil A, Taylor B, Sanderson K, Cyril S, Chan B, Hawkes AL, et al. Efficacy and feasibility of a tele-health intervention for acute coronary syndrome patients with depression: Results of the "MoodCare" randomized controlled trial. Annals of Behavioral Medicine. 2014;48(2):163-74. |
| Onuigbo, 2019 | Onuigbo, L. N., Eseadi, C., Ebifa, S., Ugwu, U. C., Onyishi, C. N., & Oyeoku, E. K. (2019). Effect of rational emotive behavior therapy program on depressive symptoms among university students with blindness in Nigeria. Journal of Rational-Emotive & Cognitive-Behavior Therapy, 37(1), 17-38. |
| Penckofer, 2012 | Penckofer SM, Ferrans C, Mumby P, Byrn M, Emanuele MA, Harrison PR, et al. A psychoeducational intervention (SWEEP) for depressed women with diabetes. Annals of Behavioral Medicine. 2012;44(2):192-206. |
| Petersen, 2014 | Petersen I, Hanass Hancock J, Bhana A, Govender K. A group-based counselling intervention for depression comorbid with HIV/AIDS using a task shifting approach in South Africa: A randomized controlled pilot study. Journal of Affective Disorders. 2014;158:78-84. |
| Pibernik, 2015 | Pibernik-Okanović M, Hermanns N, Ajduković D, Kos J, Prašek M, Šekerija M, Lovrenčić MV. Does treatment of subsyndromal depression improve depression-related and diabetes-related outcomes? A randomised controlled comparison of psychoeducation, physical exercise and enhanced treatment as usual. Trials. 2015 Jul 15;16:305. |
| Poleshuck, 2014 | Poleshuck EL, Gamble SA, Bellenger K, Lu N, Tu X, Sorensen S, et al. Randomized controlled trial of interpersonal psychotherapy versus enhanced treatment as usual for women with co-occurring depression and pelvic pain. Journal of psychosomatic research. 2014;77(4):264-72. |
| Qiu, 2013 | Qiu J, Chen W, Gao X, Xu Y, Tong H, Yang M, et al. A randomized controlled trial of group cognitive behavioral therapy for Chinese breast cancer patients with major depression. Journal of psychosomatic obstetrics and gynaecology. 2013;34(2):60-7. |
| Ransom, 2008 | Ransom D, Heckman TG, Anderson T, Garske J, Holroyd K, Basta T. Telephone-delivered, interpersonal psychotherapy for HIV-infected rural persons with depression: A pilot trial. Psychiatric Services. 2008;59(8):871-7. |
| Richards, 2018 | Richards SH, Dickens C, Anderson R, et al. Assessing the effectiveness of Enhanced Psychological Care for patients with depressive symptoms attending cardiac rehabilitation compared with treatment as usual (CADENCE): a pilot cluster randomised controlled trial. Trials 2018; 19(1). |
| Safren, 2009 | Safren SA, O'Cleirigh C, Tan JY, Raminani SR, Reilly LC, Otto MW, et al. A randomized controlled trial of cognitive behavioral therapy for adherence and depression (CBT-AD) in HIV-infected individuals. Health Psychology. 2009;28(1):1-10. |
| Safren, 2014 | Safren SA, Gonzalez JS, Wexler DJ, Psaros C, Delahanty LM, Blashill AJ, et al. A randomized controlled trial of cognitive behavioral therapy for adherence and depression (CBT-AD) in patients with uncontrolled type 2 diabetes. Diabetes care. 2014;37(3):625-33. |
| Safren, 2016 | Safren SA, Bedoya CA, O'Cleirigh C, Biello KB, Pinkston MM, Stein MD, et al. Cognitive behavioural therapy for adherence and depression in patients with HIV: A three-arm randomised controlled trial. The Lancet HIV. 2016;3(11):e529-e38. |
| Savard, 2006 | Savard J, Simard S, Giguere I, Ivers H, Morin CM, Maunsell E, et al. Randomized clinical trial on cognitive therapy for depression in women with metastatic breast cancer: Psychological and immunological effects. Palliative and Supportive Care. 2006;4(3):219-37. |
| Serfaty, 2019 | Serfaty M, King M, Nazareth I, Moorey S, Aspden T, Mannix K, et al. Effectiveness of cognitive-behavioural therapy for depression in advanced cancer: CanTalk randomised controlled trial. The British journal of psychiatry: the journal of mental science. 2019; 216, 213–221. |
| Simoni, 2013 | Simoni JM, Wiebe JS, Sauceda JA, Huh D, Sanchez G, Longoria V, et al. A preliminary RCT of CBT-AD for adherence and depression among HIV-positive Latinos on the U.S.-Mexico border: The Nuevo Dia study. AIDS and behavior. 2013;17(8):2816-29. |
| Simson, 2008 | Simson U, Nawarotzky U, Friese G, Porck W, Schottenfeld-Naor Y, Hahn S, et al. Psychotherapy intervention to reduce depressive symptoms in patients with diabetic foot syndrome. Diabetic Medicine. 2008;25(2):206-12. |
| Strong, 2008 | Strong V, Waters R, Hibberd C, Murray G, Wall L, Walker J, et al. Management of depression for people with cancer (SMaRT oncology 1): A randomised trial. Lancet. 2008;372(9632):40-8. |
| Taylor, 2009 | Taylor CB, Conrad A, Wilhelm FH, Strachowski D, Khaylis A, Neri E, et al. Does improving mood in depressed patients alter factors that may affect cardiovascular disease risk? Journal of Psychiatric Research. 2009;43(16):1246-52. |
| Teri, 1997 | Teri L, Logsdon RG, Uomoto J, McCurry SM. Behavioral treatment of depression in dementia patients: A controlled clinical trial. The journals of gerontology Series B, Psychological sciences and social sciences. 1997;52(4):P159-66. |
| Thomas, 2019 | Thomas SA, Drummond AE, Lincoln NB, Palmer RL, das Nair R, Latimer, NR, . . . Topcu G. Behavioural activation therapy for post-stroke depression: the BEADS feasibility RCT. Health technology assessment. 2019;23(47), 1-176. |
| Tovote, 2014 | Tovote KA, Fleer J, Snippe E, Peeters A, Emmelkamp PMG, Sanderman R, et al. Individual mindfulness-based cognitive therapy and cognitive behavior therapy for treating depressive symptoms in patients with diabetes: Results of a randomized controlled trial. Diabetes care. 2014;37(9):2427-34. |
| Turner, 2013 | Turner A, Hambridge J, Baker A, Bowman J, McElduff P. Randomised controlled trial of group cognitive behaviour therapy versus brief intervention for depression in cardiac patients. Australian and New Zealand Journal of Psychiatry. 2013;47(3):235-43. |
| Van Bastelaar, 2011 | Van Bastelaar KM, Pouwer F, Cuijpers P, Riper H, Snoek FJ. Web-based depression treatment for type 1 and type 2 diabetic patients: A randomized, controlled trial. Diabetes care. 2011;34(2):320-5. |
| Zhao, 2019 | Zhao Y, Munro-Kramer ML, Shi S, Wang J, Zhao Q. Effects of antenatal depression screening and intervention among Chinese high-risk pregnant women with medically defined complications: A randomized controlled trial. Early intervention in psychiatry*.* 2019; 13(5), 1090-1098. |

## **eTable 3. Characteristics of the 75 included studies**

| **Study** | **Somatic disease** | **Category** | **Country** | **Rcr** | **D_x_** | **Age** | **Wom** | **Psy** | **Frm** | **Sess** | **Ctrl** | **SG** | **AC** | **BA** | **ITT** | **SR** |
| --- | --- | --- | --- | --- | --- | --- | --- | --- | --- | --- | --- | --- | --- | --- | --- | --- |
| Abas, 2018 | HIV | HIV | Zimbabwe | Med | - | 38 | 0,66 | pst | ind | 5 | CAU | + | + | SR | + | - |
| Ahmadpanah, 2016 -3rd | Hypertension | Cardiometabolic | Iran | Med | - | 36 | 1 | 3rd | ind | 8 | CAU | + | + | SR | - | - |
| Ahmadpanah, 2016 -str | Hypertension | Cardiometabolic | Iran | Med | - | 36 | 1 | Oth | grp | 8 | CAU | + | + | SR | - | - |
| Bedard, 2014 | Traumatic brain injury | Other | Canada | Com | - | 46 | 0,45 | 3rd | grp | 10 | WL | + | + | SR | - | - |
| Beutel, 2014 | Breast cancer | Oncological | Germany | Med | + | 52 | 1 | dyn | ind | 18 | CAU | + | + | + | + | - |
| Boele, 2018 | Glioma | Oncological | NL | Com | - | 45 | 0,55 | pst | gsh | 5 | WL | + | + | SR | + | + |
| Boeschoten, 2017 | Multiple sclerosis | Neurological | NL | Com | - | 49 | 0,8 | pst | gsh | 5 | WL | + | + | SR | + | + |
| Buhrman, 2015 | Chronic pain | Other | Sweden | Com | - | 51 | 0,85 | cbt | gsh | 6 | WL | + | + | SR | + | - |
| Burns, 2007 | Surgery for hip fracture | Other | UK | Med | - | 81 | 0,77 | oth | ind | 6 | CAU | + | + | + | + | - |
| Chesney, 2003 | HIV | HIV | US | Com | - | 39 | 0 | oth | grp | 10 | oth | - | - | SR | - | - |
| De Groot, 2019 -cbt | Diabetes | Cardiometabolic | US | Com | + | 56 | 0,77 | cbt | ind | 10 | CAU | + | + | + | + | - |
| De Groot, 2019 -cbt + exer | Diabetes | Cardiometabolic | US | Com | + | 56 | 0,77 | cbt | ind | 22 | CAU | + | + | + | + | - |
| De Jong, 2018 | Chronic pain | Other | US | Com | + | 51 | 0,75 | 3rd | grp | 7 | WL | - | - | - | + | + |
| Dekker, 2012 | Heart failure | Cardiometabolic | US | Med | - | 66 | 0,74 | cbt | ind | 1 | CAU | + | + | SR | + | - |
| Desautels, 2017 | Breast cancer | Oncological | Canada | Med | - | 57 | 1 | cbt | ind | 8 | WL | + | + | + | + | - |
| Dindo, 2012 | Migraine | Neurological | US | Med | + | 33 | 0,93 | 3rd | grp | 1 | WL | - | - | - | + | - |
| Dindo, 2019 | Migraine | Neurological | US | Com | + | 36 | 0,83 | 3rd | grp | 1 | oth | - | - | + | + | - |
| Dobkin, 2011 | Parkinson’s Disease | Neurological | US | Com | + | 65 | 0,4 | cbt | ind | 10 | CAU | + | - | + | + | - |
| Doering, 2013 | Recovering from cardiac surgery | Cardiometabolic | US | Med | + | 64 | 0,31 | cbt | ind | 8 | CAU | + | - | SR | + | - |
| Dong, 2019 | Colorectal cancer | Oncological | China | Med | - | 59 | 0,5 | lrt | tele | 6 | oth | + | + | + | + | - |
| Duarte, 2009 | End-stage renal disease | Other | Brazil | Med | + | 53 | 0,59 | cbt | grp | 12 | CAU | - | + | SR | - | - |
| Evans, 1995 - cbt | Cancer | Oncological | US | Med | - | 54 | 0,35 | cbt | grp | 8 | CAU | - | - | SR | - | - |
| Evans, 1995 - sup | Cancer | Oncological | US | Med | - | 54 | 0,35 | sup | grp | 8 | CAU | - | - | SR | - | - |
| Fann, 2015 - ind | Traumatic Brain Injury | Other | US | Com | + | 46 | 0,37 | cbt | tele | 10 | CAU | + | - | + | - | - |
| Fann, 2015 - tel | Traumatic Brain Injury | Other | US | Com | + | 46 | 0,37 | cbt | ind | 9 | CAU | + | - | + | - | - |
| Freedland, 2009 -cbt | Coronary artery bypass surgery | Cardiometabolic | US | Med | + | 61 | 0,5 | cbt | ind | 11 | CAU | + | + | + | + | - |
| Freedland, 2009 -ssm | Coronary artery bypass surgery | Cardiometabolic | US | Med | + | 61 | 0,5 | sup | ind | 8 | CAU | + | + | + | + | - |
| Freedland, 2015 | Heart failure | Cardiometabolic | US | Med | + | 56 | 0,46 | cbt | ind | 11 | CAU | + | + | + | + | + |
| Gellis, 2008 | Cardiovascular disease | Cardiometabolic | US | Med | - | 77 | 0,87 | pst | ind | 6 | CAU | + | - | + | - | - |
| Gellis, 2010 | Medically ill home care patients | Other | US | Med | - | 76 | 0,92 | pst | ind | 6 | CAU | - | + | + | - | - |
| Heckman, 2011 -cop | HIV | HIV | US | Com | - | 55 | 0,33 | oth | grp | 7 | CAU | + | - | SR | + | - |
| Heckman, 2011 -sup | HIV | HIV | US | Com | - | 55 | 0,33 | sup | grp | 7 | CAU | + | - | SR | + | - |
| Heckman, 2013 -cop | HIV | HIV | US | Med | - | 59 | 0,39 | oth | oth | 6 | CAU | + | - | SR | + | - |
| Heckman, 2013 -sup | HIV | HIV | US | Med | - | 59 | 0,39 | sup | oth | 7 | CAU | + | - | SR | + | - |
| Heckman, 2017 | HIV | HIV | US | Com | + | 52 | 0,37 | ipt | tele | 8 | CAU | + | - | SR | + | - |
| Hermanns, 2015 | Diabetes | Cardiometabolic | Germany | Med | - | 43 | 0,57 | cbt | grp | 5 | CAU | - | + | SR | + | - |
| Herrmann-Lingen, 2016 | Coronary artery disease | Cardiometabolic | Germany | Med | - | 59 | 0,21 | dyn | oth | 16 | CAU | + | - | + | + | + |
| Huang, 2016 | Diabetes | Cardiometabolic | Taiwan | Med | - | 56 | 0,52 | cbt | grp | 12 | CAU | - | - | SR | - | - |
| Hum, 2019 | Epilepsy | Neurological | Canada | Com | - | 36 | 0,71 | 3rd | oth | 8 | oth | + | - | SR | + | - |
| Hummel, 2017 | Hospitalized for acute illness | Other | Germany | Med | - | 82 | 0,8 | cbt | grp | 13 | WL | + | - | + | + | - |
| Jalali, 2019 | HIV | HIV | Iran | Oth | - | 32 | 0 | oth | grp | 11 | WL | - | - | SR | - | - |
| Kamga, 2017 | Age-related eye disease | Oth | Canada | Med | - | 76 | 0,63 | cbt | gsh | 8 | WL | + | + | SR | + | + |
| Kelly, 1993 - cbt | HIV | HIV | US | Com | - | 34 | 0 | cbt | grp | 8 | CAU | - | - | SR | - | - |
| Kelly, 1993 - sup | HIV | HIV | US | Com | - | 34 | 0 | sup | grp | 8 | CAU | - | - | SR | - | - |
| Kim, 2018 | Breast cancer | Oncological | Korea | Med | - | 48 | 1 | cbt | oth | 7 | CAU | + | - | SR | - | - |
| Lamers, 2010 | Chronic illnesses | Other | NL | Med | + | 71 | 0,47 | cbt | ind | 4 | CAU | + | + | SR | + | - |
| Larcombe, 1984 | Multiple sclerosis | Neurological | Australia | Com | + | 43 | 0,68 | cbt | grp | 6 | WL | - | - | + | - | - |
| Lloyd-Williams, 2018 | Cancer (palliative care) | Oncological | UK | Med | - | 65 | 0,71 | oth | ind | 1 | CAU | - | + | SR | + | - |
| Lök, 2019 | Alzheimer | Neurological | Turkey | Med | - | NR | 0,57 | lrt | grp | 8 | CAU | + | + | - | + | - |
| Lundgren, 2016 | Heart failure | Cardiometabolic | Sweden | Med | - | 63 | 0,42 | cbt | gsh | 7 | oth | + | + | SR | + | + |
| Lustman, 1998 | Diabetes | Cardiometabolic | US | Com | + | 55 | 0,6 | cbt | ind | 10 | CAU | + | + | SR | - | - |
| Martin, 2015 | Migraine or tension-type headache | Neurological | Australia | Com | + | 41 | 0,74 | cbt | ind | 12 | WL | + | + | SR | - | - |
| Mohr, 2000 | Multiple sclerosis | Neurological | US | Med | - | 42 | 0,72 | cbt | tele | 8 | CAU | - | - | SR | + | - |
| Mossey, 1996 | Hospitalized for acute illness | Other | US | Med | - | 71 | 0,78 | ipt | ind | 10 | CAU | - | - | SR | - | - |
| Nakimuli, 2015 | HIV | HIV | Uganda | Med | + | 44 | 0,42 | sup | grp | 8 | oth | + | + | SR | + | - |
| Newby, 2017 | Diabetes | Cardiometabolic | Australia | Com | + | 47 | 0,71 | cbt | gsh | 6 | WL | + | + | SR | + | + |
| Nobis, 2015 | Diabetes | Cardiometabolic | Germany | Com | - | 51 | 0,63 | oth | gsh | 6 | oth | + | - | SR | + | + |
| Nollett, 2016 | Visual impairment | Other | UK | Med | - | 70 | 0,59 | pst | ind | 6 | CAU | + | + | SR | - | - |
| Olukolade, 2017 | Stroke survivors | Cardiometabolic | Nigeria | Med | - | NR | 0,57 | cbt | ind | 9 | WL | + | - | SR | - | - |
| O'Neil, 2014 | Acute coronary syndrome | Cardiometabolic | Australia | Med | - | 60 | 0,25 | cbt | tele | 8 | CAU | + | + | + | + | - |
| Onuigbo, 2019 | Blindness | Other | Nigeria | Med | - | 25 | 0,54 | cbt | grp | 14 | CAU | + | + | SR | + | - |
| Penckofer, 2012 | Diabetes | Cardiometabolic | US | Com | - | 54 | 1 | cbt | grp | 8 | CAU | + | - | SR | + | - |
| Petersen, 2014 | HIV/AIDS | HIV | South Africa | Med | + | 37 | 0,74 | ipt | grp | 8 | CAU | + | - | SR | - | - |
| Pibernik, 2015 | Diabetes | Cardiometabolic | Croatia | Med | - | 58 | 0,54 | cbt | grp | 6 | CAU | + | + | SR | + | - |
| Poleshuck, 2014 | Chronic pelvic pain | Other | US | Med | + | 37 | 1 | ipt | ind | 4 | CAU | + | - | - | + | - |
| Qiu, 2013 | Breast cancer | Oncological | China | Med | + | 51 | 1 | cbt | grp | 10 | WL | + | + | + | + | - |
| Ransom, 2008 | HIV/AIDS | HIV | US | Com | + | 44 | 0,16 | ipt | tele | 6 | CAU | - | - | SR | + | - |
| Richards, 2018 | Coronary heart disease | Cardiometabolic | UK | Med | - | 65 | 0,48 | bat | ind | 8 | CAU | + | + | SR | + | + |
| Safren, 2009 | HIV | HIV | US | Com | + | NR | 0,16 | cbt | ind | 10 | CAU | - | - | + | + | - |
| Safren, 2014 | Diabetes | Cardiometabolic | US | Med | + | 57 | 0,49 | cbt | ind | 10 | CAU | + | + | + | - | - |
| Safren, 2016 -cbt | HIV | HIV | US | Com | + | 47 | 0,31 | cbt | ind | 11 | CAU | + | - | + | + | - |
| Safren, 2016 -sup | HIV | HIV | US | Com | + | 47 | 0,31 | sup | ind | 11 | CAU | + | - | + | + | - |
| Savard, 2006 | Breast cancer | Oncological | Canada | Com | - | 52 | 1 | cbt | ind | 8 | WL | + | + | + | - | - |
| Serfaty, 2019 | Advanced cancer | Oncological | UK | Med | + | 60 | 0,66 | cbt | ind | 5 | CAU | + | + | SR | + | + |
| Simoni, 2013 | HIV | HIV | US | Com | - | 46 | 0,28 | cbt | ind | 11 | CAU | + | + | + | + | - |
| Simson, 2008 | Diabetic foot syndrome | Cardiometabolic | Germany | Med | - | 61 | 0,43 | sup | ind | 5 | CAU | - | - | SR | + | - |
| Strong, 2008 | Cancer (various) | Oncological | UK | Med | + | 57 | 0,71 | pst | ind | 10 | CAU | + | + | SR | + | - |
| Taylor, 2009 | High risk for coronary artery disease | Cardiometabolic | US | Com | - | 62 | 0,67 | cbt | ind | 15 | WL | - | - | + | + | - |
| Teri, 1997 - ba | Dementia | Neurological | US | Med | + | 76 | 0,47 | bat | oth | 9 | CAU | - | - | + | - | - |
| Teri, 1997 - pst | Dementia | Neurological | US | Med | + | 76 | 0,47 | pst | oth | 9 | CAU | - | - | + | - | - |
| Thomas, 2019 | Post-stroke depression | Cardiometabolic | UK | Com | - | 66 | 39,6 | bat | ind | 8 | CAU | + | + | + | + | + |
| Tovote, 2014 - cbt | Diabetes | Cardiometabolic | NL | Med | - | 53 | 0,49 | 3rd | ind | 8 | WL | + | - | - | + | - |
| Tovote, 2014 - mbct | Diabetes | Cardiometabolic | NL | Med | - | 53 | 0,49 | cbt | ind | 8 | WL | + | - | - | + | - |
| Turner, 2013 | Cardiac patients | Cardiometabolic | Australia | Com | - | 62 | 0,26 | cbt | grp | 6 | oth | + | + | SR | - | - |
| Van Bastelaar, 2011 | Diabetes | Cardiometabolic | NL | Com | - | 50 | 0,61 | cbt | gsh | 8 | WL | + | + | SR | + | - |
| Zhao, 2019 | Pregnant with medical complications | Other | China | Med | - | 31 | 1 | oth | grp | 4 | CAU | + | - | SR | - | - |

Abbreviations: Rcr: recruitment (med: medical settings; Com: community); D_x_: diagnostic interview at baseline (+: yes; -: no); Age: Mean age; Wom: proportion of women; NR: not reported; Psy: type of therapy (cbt: cognitive behavior therapy; pst: problem-solving therapy; 3rd: third wave therapy; dyn: psychodynamic therapy; ipt: interpersonal psychotherapy; bat: behavioral activation therapy; sup: non-directive supportive therapy; lrt: life review therapy; oth: other type of therapy). Frm: format (ind: individual; grp: group; gsh: guided self-help; oth: other/mixed format); Sess: Average number of sessions received; Ctrl: type of control condition (WL: waiting list; CAU: care as usual; oth: other type of inactive control); SG: sequence generation, rated as + positive or - negative (negative includes unclear); AC: allocation concealment; BA: blinded assessment; ITT: intention to treat analyses; SR: Selective reporting.

## **Effects of psychotherapy on depression severity**

###
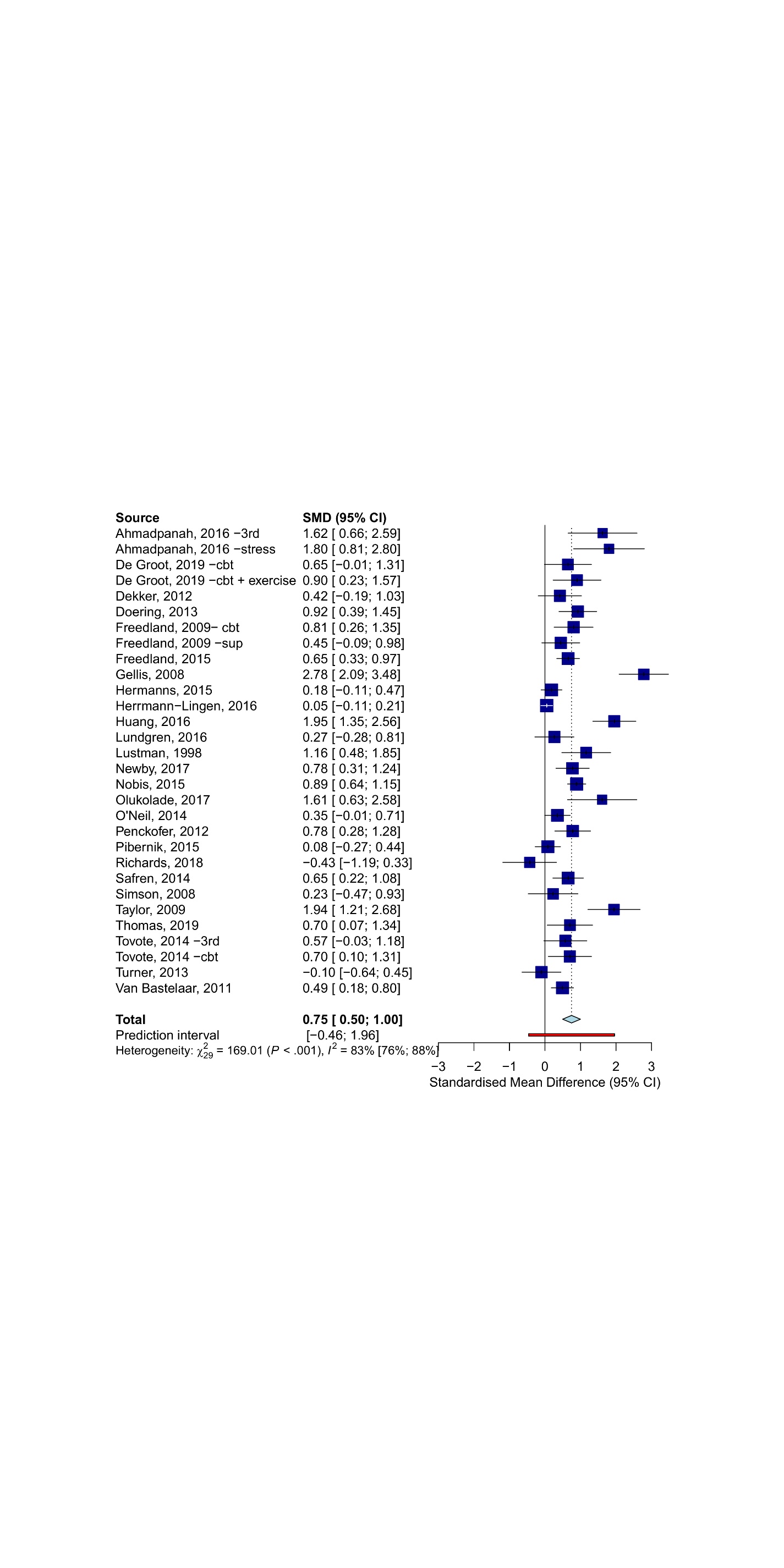
eFigure 1. Cardiometabolic disorders

### eFigure 2. Oncological disorders


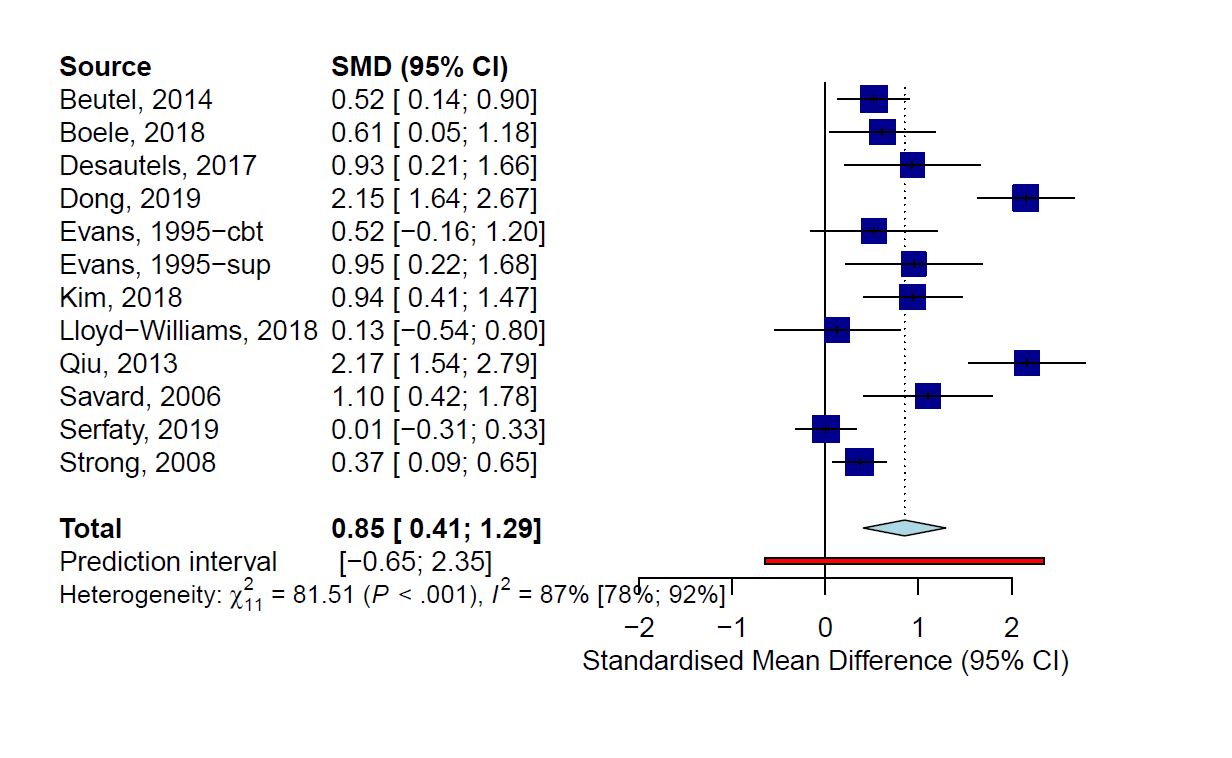


### eFigure 3. HIV/AIDS


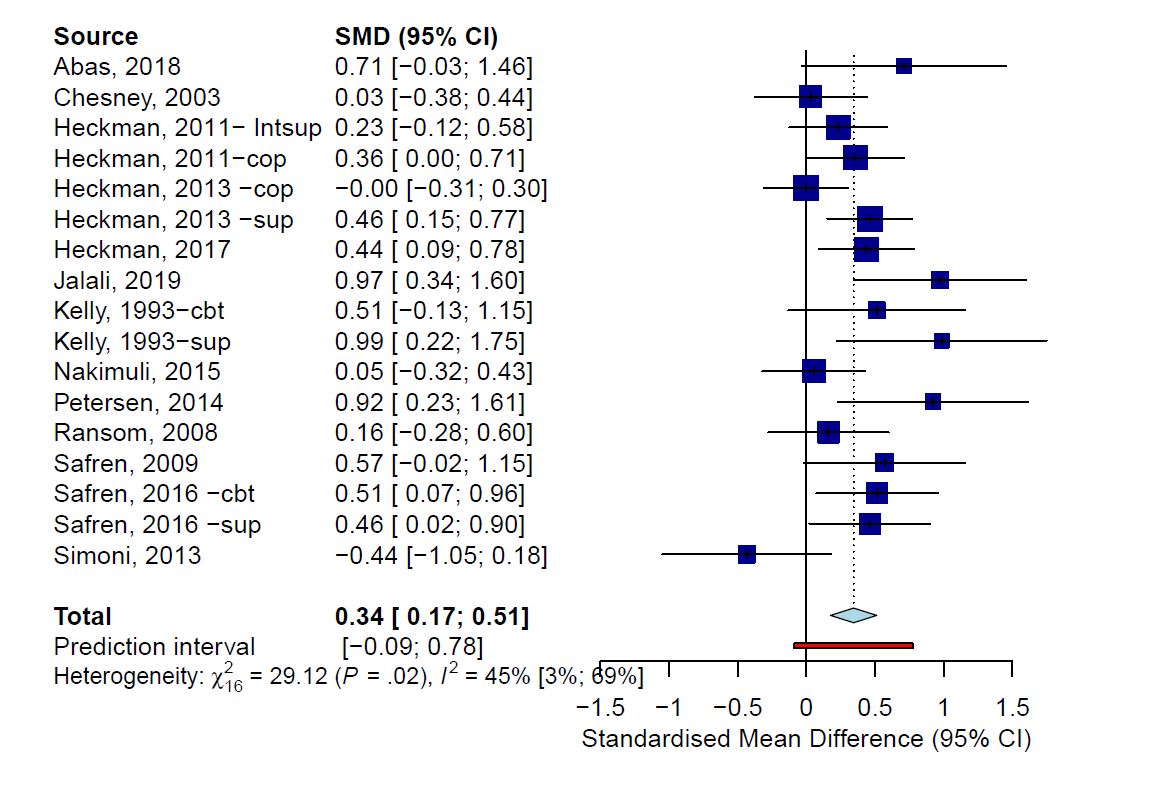


### eFigure 4. Neurological disorders


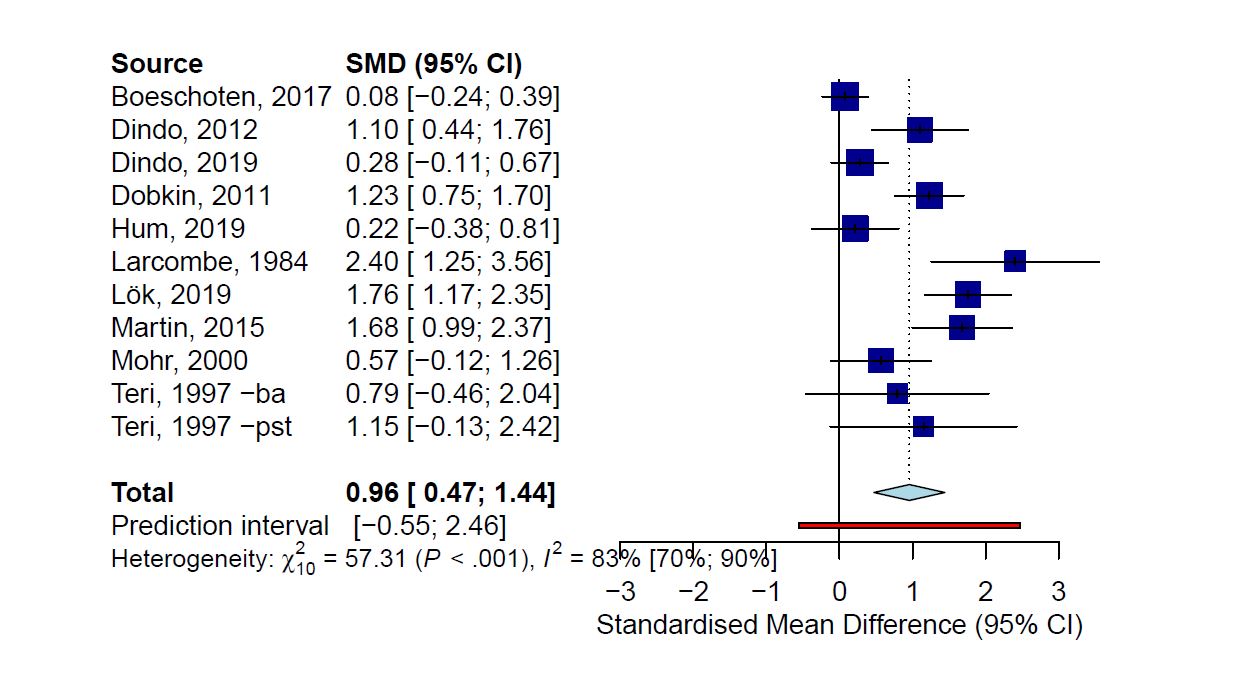


### eFigure 5. Other somatic disorders


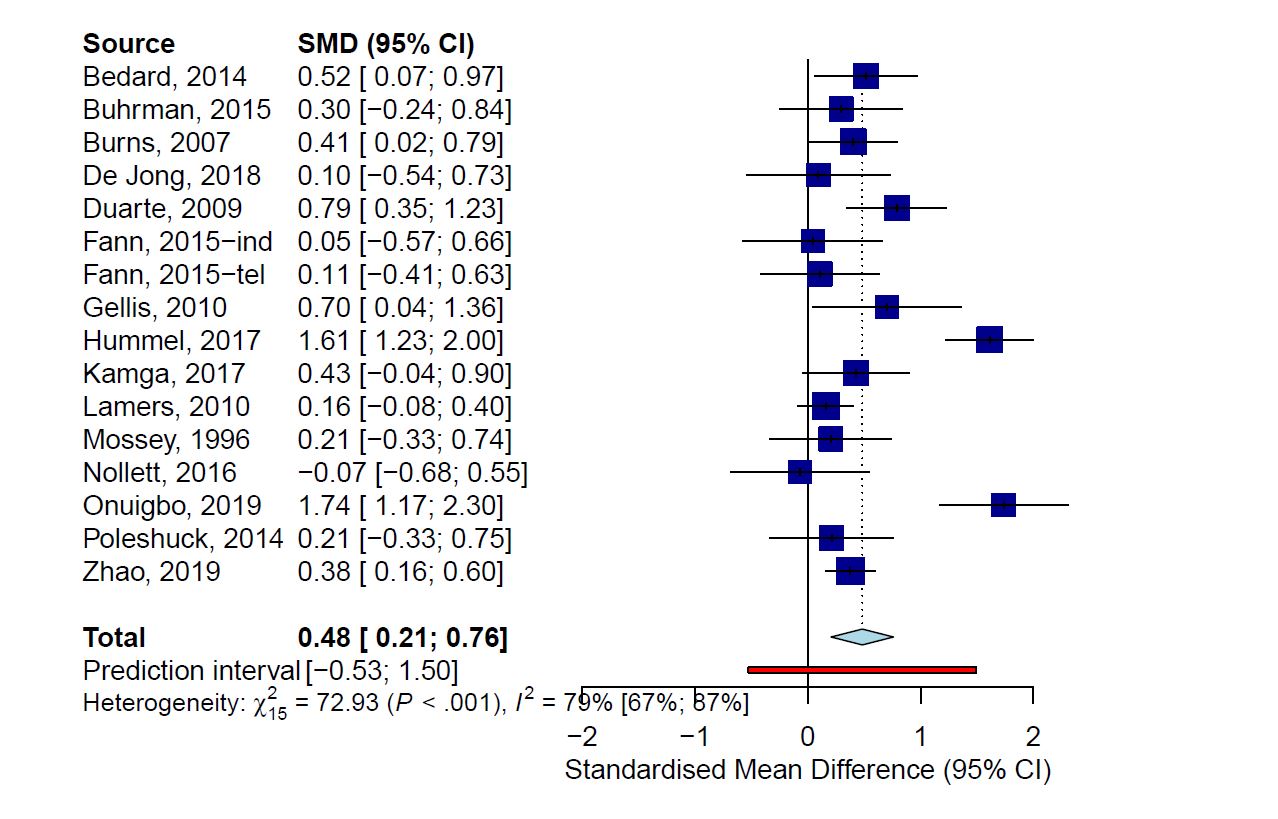


### Long term outcomes on depression severity

|  | N_comp_ | *g* | 95% CI | *I^2^* | 95% CI |
| --- | --- | --- | --- | --- | --- |
| ≥ 6 months post-randomization | 59 | 0.32 | 0.19, 0.44 | 73 | 65, 79 |
| Outliers excluded | 53 | 0.25 | 0.18, 0.31 | 35 | 9, 54 |
| Studies at low RoB | 26 | 0.30 | 0.15, 0.46 | 75 | 63, 83 |
| Adjusted for publication bias | 59 | 0.32 | 0.19, 0.44 | 73 | 65, 79 |
| **Somatic disorders** |  |  |  |  |  |
| Cardiometabolic | 24 | 0.41 | 0.14, 0.68 | 85 | 70, 90 |
| Oncological | 6 | 0.49 | 0.08, 0.90 | 76 | 46, 89 |
| HIV/AIDS | 17 | 0.27 | 0.17, 0.37 | 12 | 0, 49 |
| Neurological | 3 | 0.12 | -0.24, 0.49 | 41 | 0, 82 |
| Other | 9 | 0.23 | 0.10, 0.36 | 0 | 0, 59 |
| 6- 11 months post-randomization | 46 | 0.38 | 0.22, 0.53 | 75 | 67, 81 |
| ≥ 12 months post-randomization | 13 | 0.13 | 0.04, 0.21 | 0 | 0, 57 |

## **Effects of psychotherapy on Quality of life**

### Additional analyses on Quality of life outcomes

|  | N_comp_ | *g* | 95% CI | *I^2^* | 95% CI | *p-*value^*^ |
| --- | --- | --- | --- | --- | --- | --- |
| **Overall QoL** | 40 | 0.26 | 0.17, 0.35 | 34 | 3, 56 | NA |
| Physical QoL | 23 | 0.22 | 0.11, 0.34 | 32 | 0, 59 | NA |
| Mental QoL | 24 | 0.46 | 0.34, 0.57 | 36 | 0, 61 | NA |
| Outliers excluded | 39 | 0.24 | 0.16, 0.32 | 23 | 0, 48 | NA |
| Studies at low RoB | 23 | 0.23 | 0.14, 0.33 | 9 | 0, 43 | NA |
| Adjusted for publication bias | 46 | 0.19 | 0.09, 0.29 | 54 | 36, 67 | NA |
| **Somatic disorders** | | | | | | |
| Cardiometabolic | 17 | 0.22 | 0.12, 0.32 | 0 | 0, 50 | 0.763 |
| Oncological | 7 | 0.43 | 0.14, 0.71 | 66 | 23, 85 |  |
| HIV/AIDS | 8 | 0.26 | 0.09, 0.43 | 27 | 0, 67 |  |
| Neurological | 7 | 0.23 | -0.03, 0.49 | 61 | 11, 83 |  |
| Other | 17 | 0.22 | 0.12, 0.32 | 0 | 0, 50 |  |
| **Subgroup analyses** |  |  |  |  |  |  |
| Age group |  |  |  |  |  |  |
| Adults | 18 | 0.24 | 0.13, 0.35 | 16 | 0, 51 | 0.586 |
| Older adults | 22 | 0.28 | 0.15, 0.42 | 46 | 11, 67 |  |
| Recruitment |  |  |  |  |  |  |
| Community | 15 | 0.22 | 0.08, 0.37 | 27 | 0, 61 | 0.489 |
| Medical settings | 25 | 0.29 | 0.18, 0.40 | 40 | 3, 63 |  |
| Diagnosis of depression |  |  |  |  |  |  |
| Confirmed diagnosis | 18 | 0.28 | 0.19, 0.38 | 0 | 0, 48 | 0.486 |
| Elevated symptoms | 22 | 0.22 | 0.09, 0.36 | 51 | 19, 70 |  |
| Type of psychotherapy |  |  |  |  |  |  |
| CBT | 22 | 0.29 | 0.18, 0.40 | 32 | 0, 59 | 0.015 |
| PST | 6 | 0.02 | -0.13, 0.17 | 0 | 0, 67 |  |
| 3^rd^ wave | 4 | 0.32 | -0.01, 0.65 | 30 | 0, 75 |  |
| Other | 8 | 0.36 | 0.16, 0.55 | 37 | 0, 72 |  |
| Format |  |  |  |  |  |  |
| Individual | 18 | 0.21 | 0.09, 0.32 | 16 | 0, 52 | 0.574 |
| Group | 12 | 0.32 | 0.14, 0.50 | 55 | 14, 77 |  |
| Guided self-help | 6 | 0.22 | 0.02, 0.43 | 40 | 0, 76 |  |
| Type of control |  |  |  |  |  |  |
| Usual care | 25 | 0.22 | 0.12, 0.32 | 23 | 0, 53 | 0.188 |
| Waiting list | 9 | 0.29 | 0.05, 0.53 | 62 | 23, 82 |  |
| Other | 6 | 0.37 | 0.24, 0.50 | 0 | 0, 65 |  |
| Country |  |  |  |  |  |  |
| Western | 33 | 0.23 | 0.14, 0.33 | 34 | 0, 57 | 0.129 |
| Non-Western | 7 | 0.40 | 0.21, ,0.60 | 23 | 0, 66 |  |

^*^The *P* values indicate whether the difference between the effect sizes in the subgroups is significant.

Abbreviations: N_comp_, Number of comparisons; *g*, Hedges’ g; QoL: Quality of life; CBT, cognitive behavioral therapy; PST, problem-solving therapy; 3^rd^ Wave: Third wave therapies; NA, not applicable; RoB: Risk of bias.

###
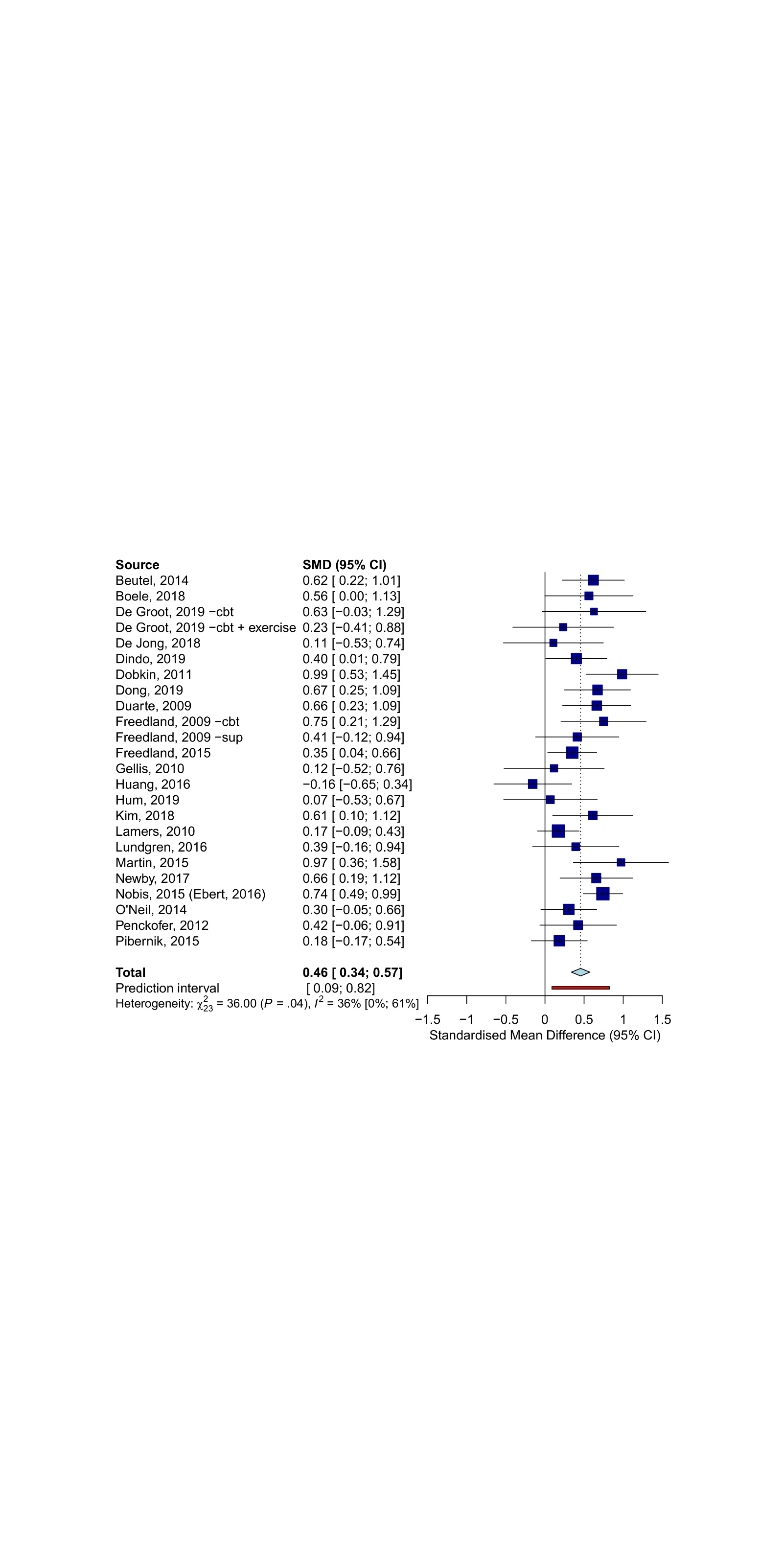
eFigure 6. Forest plot Mental Quality of life

### eFigure 7. Forest plot Physical Quality of life


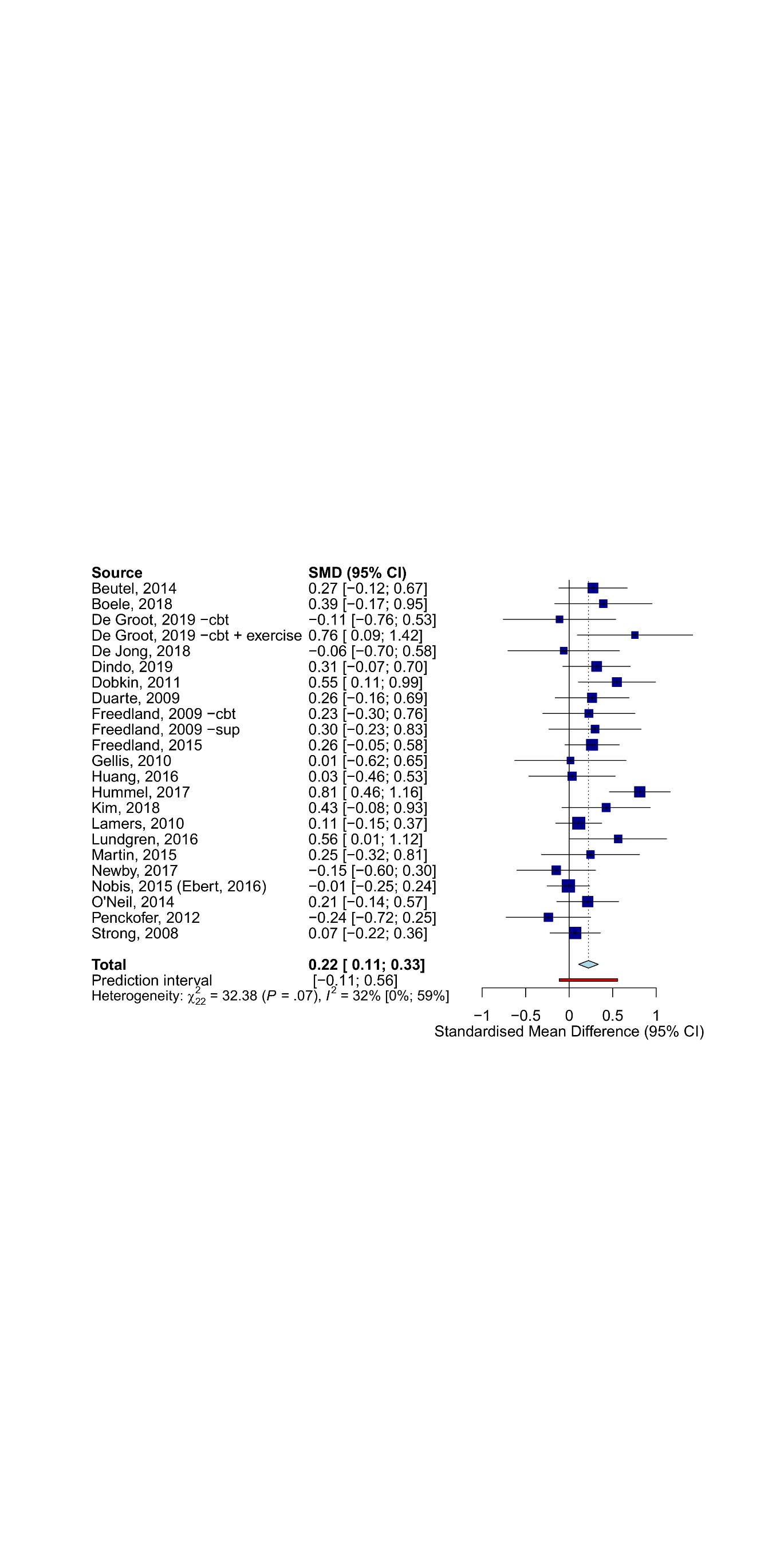


### Long term outcomes

|  | N_comp_ | *g* | 95% CI | *I^2^* | 95% CI |
| --- | --- | --- | --- | --- | --- |
| ≥ 6 months post-randomization |  |  |  |  |  |
| Overall QoL | 25 | 0.23 | 0.15, 0.30 | 0 | 0, 37 |
| Mental QoL | 18 | 0.34 | 0.22, 0.45 | 29 | 0, 60 |
| Physical QoL | 15 | 0.15 | 0.08, 0.23 | 0 | 0, 9 |
| Outliers excluded | 24 | 0.22 | 0.15, 0.28 | 0 | 0, 20 |
| Studies at low RoB | 16 | 0.26 | 0.20, 0.31 | 0 | 0, 0 |
| Adjusted for publication bias | 25 | 0.23 | 0.15, 0.30 | 0 | 0, 37 |
| **Somatic disorders** |  |  |  |  |  |
| Cardiometabolic | 15 | 0.22 | 0.15, 0.29 | 0 | 0, 13 |
| All other somatic disorders | 10 | 0.25 | 0.09, 0.40 | 34 | 0, 69 |
|  |  |  |  |  |  |
| 6- 11 months post-randomization | 12 | 0.25 | 0.16, 0.34 | 0 | 0, 2 |
| ≥ 12 months post-randomization | 5 | 0.12 | -0.12, 0.37 | 24 | 0, 69 |

Abbreviations: N_comp_, Number of comparisons; *g*, Hedges’ g; QoL: Quality of life; RoB: Risk of bias.

# **Effects of psychotherapy on somatic health-related outcomes and mortality**

## **eFigure 8. Forest plot of Glycaemic control**


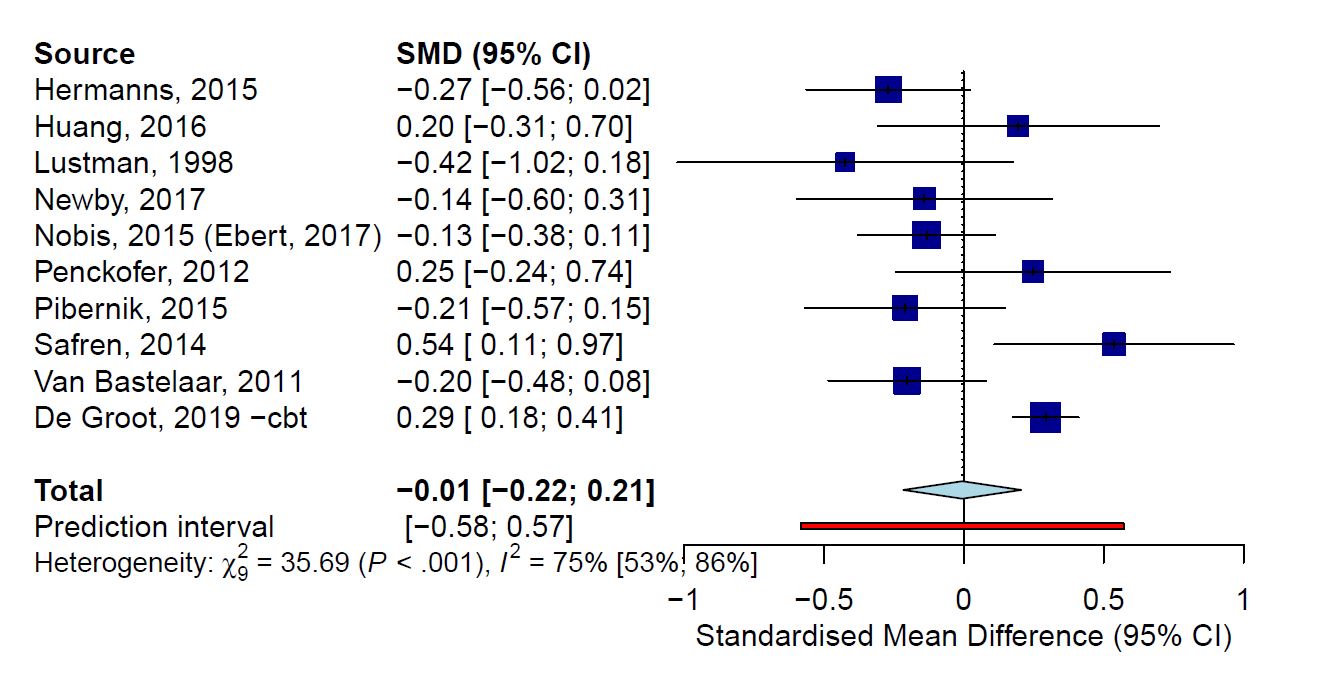


## **eFigure 9. Forest plot of Pain outcomes**


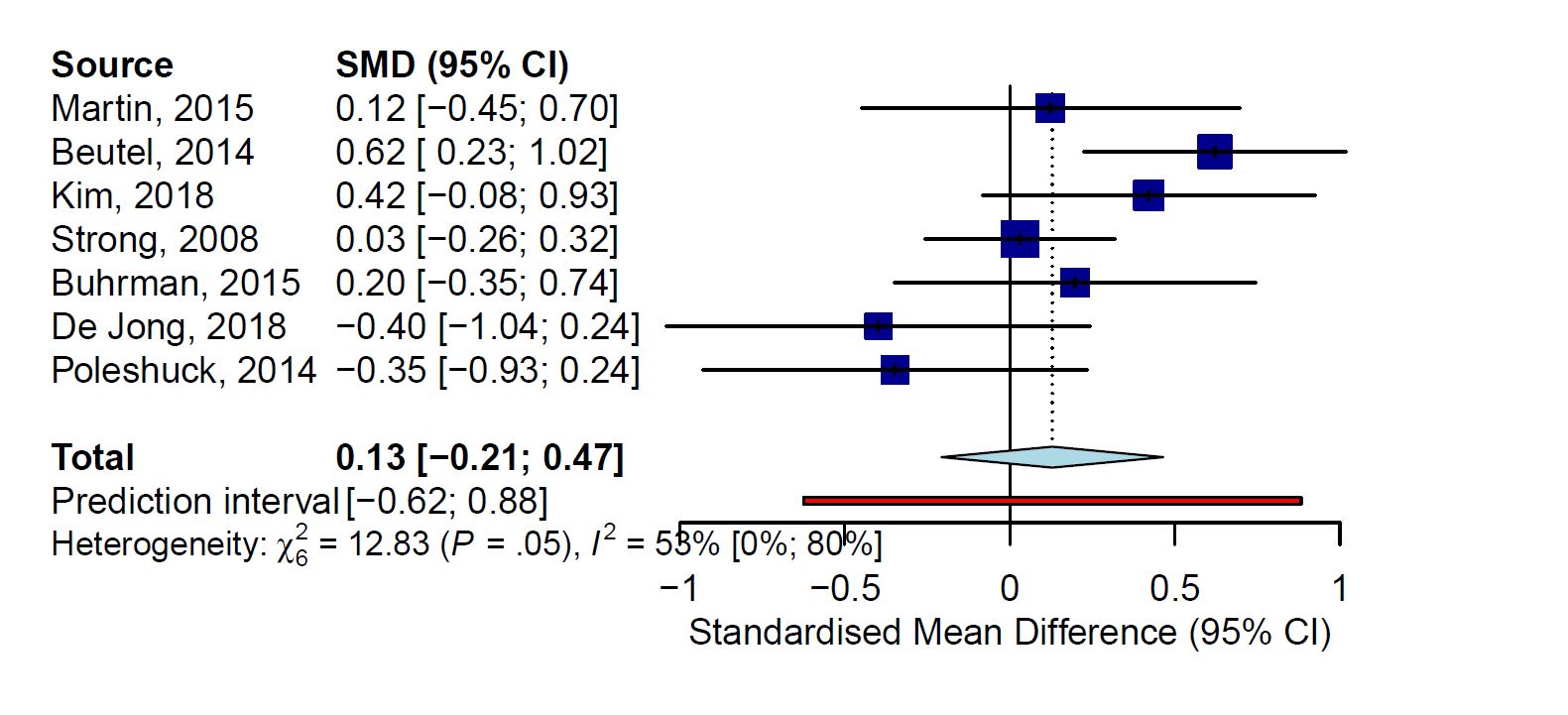


## **eFigure 10. Forest plot of Mortality**


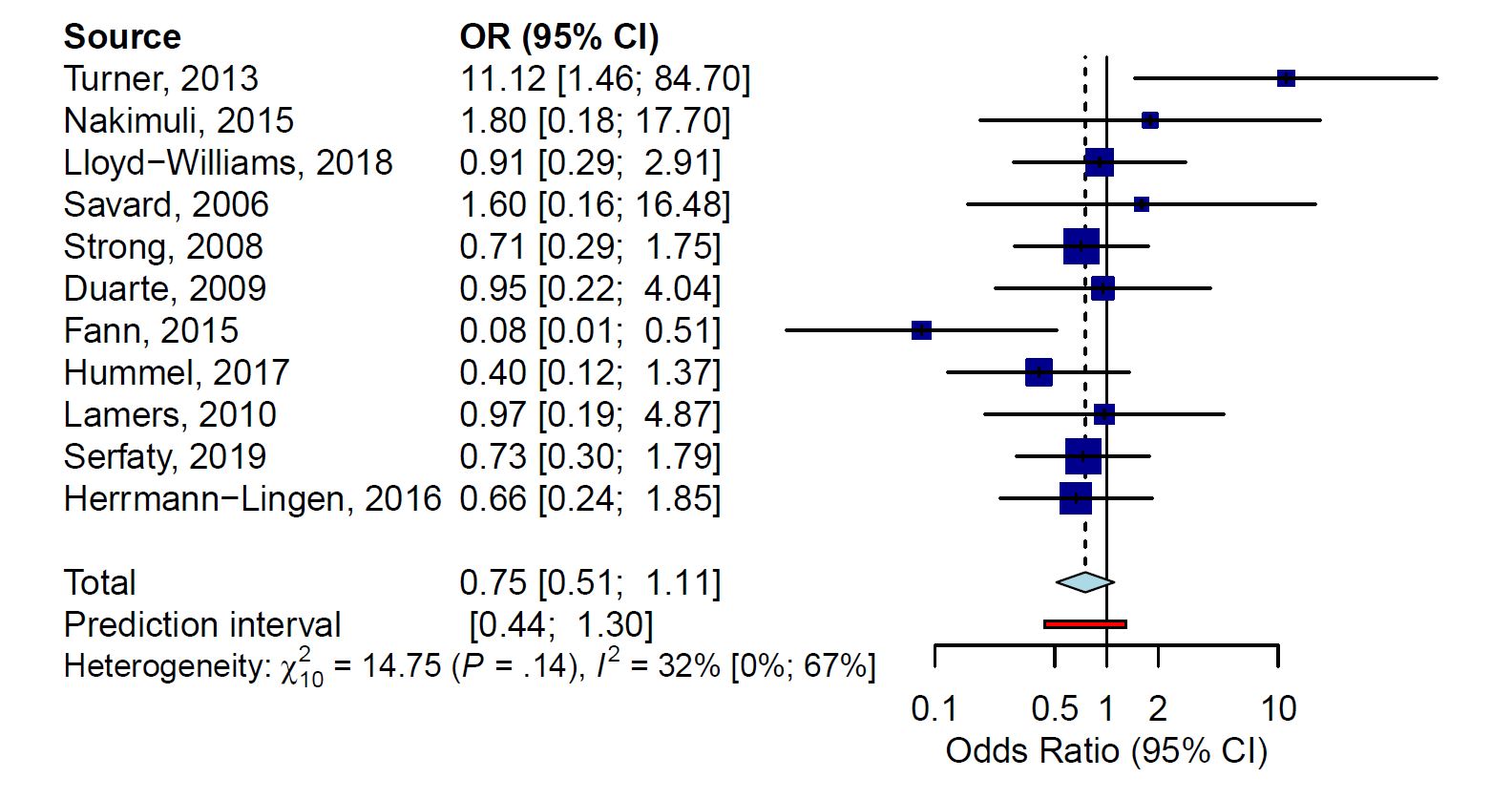

Supplement: Supplementary file 1 [file S0033291721004414sup001.docx]
